# Exploring the structural basis and functional immunodynamics of immunoglobulin M in host defense against fungal pathogens

**DOI:** 10.3389/fimmu.2025.1666690

**Published:** 2025-10-30

**Authors:** Kritsada Pruksaphon, Nopawit Khamto, Artid Amsri, Monsicha Wagatsuma, Joshua D. Nosanchuk, Yujiro Higuchi, Sirida Youngchim

**Affiliations:** ^1^ Department of Medical Technology, School of Allied Health Sciences, Walailak University, Nakhon Si Thammarat, Thailand; ^2^ Center of Excellence Research for Melioidosis and Microorganisms (CERMM), Walailak University, Nakhon Si Thammarat, Thailand; ^3^ Department of Biochemistry, Faculty of Medical Science, Naresuan University, Phitsanulok, Thailand; ^4^ Office of Research Administration, Chiang Mai University, Chiang Mai, Thailand; ^5^ Department of Microbiology, Faculty of Medicine, Chiang Mai University, Chiang Mai, Thailand; ^6^ Bachelor Degree in Medical Technology (International Program), School of Allied Health Sciences, Walailak University, Nakhon Si Thammarat, Thailand; ^7^ Department of Medicine (Division of Infectious Diseases) and Department of Microbiology and Immunology, Albert Einstein College of Medicine, Bronx, NY, United States; ^8^ Department of Bioscience and Biotechnology, Faculty of Agriculture, Kyushu University, Fukuoka, Japan

**Keywords:** immunoglobulin M (IgM), lectins, fungal immunology, molecular docking, glycan recognitions

## Abstract

The rising prevalence of life-threatening fungal infections, particularly in immunocompromised individuals, necessitates a deeper understanding of all facets of the host immune response. While much focus has been placed on cellular immunity, the contribution of Immunoglobulin M (IgM), the first antibody produced during an immune response, remains a relatively underexplored area in the context of systemic mycoses. This comprehensive survey explores the role of IgM in antifungal immunity, with a focus on life-threatening fungal infections. As the earliest antibody isotype, IgM achieves remarkable binding diversity through germline-encoded V(D)J recombination without requiring somatic hypermutation. Its multimeric structure enables high-avidity recognition of fungal cell wall components, facilitating binding despite antigenic variability of opportunistic pathogens. While conserved fungal polysaccharides activate host pattern recognition receptors, pathogenic fungi have evolved exopolysaccharides that shield immunogenic motifs from detection. IgM recognizes these complex carbohydrate epitopes and triggers complement activation, enhancing opsonophagocytic clearance. Evolutionarily conserved across vertebrates, IgM provides critical broad-spectrum protection through germline-encoded diversity. Despite these advantages, IgM’s therapeutic potential in systemic mycoses remains underexplored, particularly in immunocompromised individuals. This review presents evidence on the molecular basis and immunological functions of IgM, highlighting its contributions to immunity against pathogenic fungi and identifying promising avenues for translational research across various clinically relevant fungal species.

## 1 Introduction

A defining feature of many immune-related molecules, especially immunoglobulins, is their remarkable binding diversity. In the acquired immune system, inherited germline variability provides the foundational elements for somatic gene-rearrangement processes that generate an immense repertoire of antigen-specific receptors. This molecular heterogeneity is indispensable for recognizing the vast and ever-changing array of antigens individuals encounter over a lifetime. Moreover, such extensive pre-immune diversity confers a profound evolutionary advantage. It ensures that even novel or rapidly mutating pathogen epitopes can be detected by at least a subset of naïve B cells. The conservation of immunoglobulin gene families and their diversification mechanisms across vertebrates highlights their fundamental role in host defense. Through affinity maturation and the establishment of immunological memory, this combinatorial and hypermutation-driven diversity supports both potent primary responses and the rapid, high-affinity secondary responses characteristic of durable immunity. Germline-encoded frameworks combined with somatic diversification therefore furnish vertebrates with the necessary breadth and precision for effective pathogen recognition and protection (1).

In the context of immunoglobulin M (IgM), this generative architecture is particularly evident as the first isotype expressed by naive B cells, IgM receptors arise directly from unmutated V(D)J recombination, augmented by junctional diversity but lacking somatic hypermutation. Its pentameric structure amplifies the binding valency of these germline-encoded specificities, enabling even low-affinity germline receptors to achieve high overall avidity. Consequently, IgM exemplifies how inherited recombinatorial diversity, without affinity maturation, can furnish broad-spectrum recognition—providing critical early defense and laying the groundwork for subsequent isotype-switched, affinity-matured immunoglobulins. IgM represents the earliest antibody isotype expressed during B lymphocyte ontogeny and serves as the initial mediator of the humoral immune response. This function is evolutionarily conserved across diverse species, ranging from jawed vertebrates (Gnathostomata) to mammals and humans (2, 3). The monomeric configuration of IgM is displayed on the surface of B lymphocytes, where it functions as the B-cell antigen receptor (BCR). Once secreted, IgM predominantly assembles into a pentamer incorporating the joining (J) chain, thereby forming polymeric, or secretory, IgM (sIgM). The J chain mediates transcytosis of these polymeric IgM molecules across mucosal epithelial barriers via interaction with the polymeric immunoglobulin receptor (pIgR), resulting in the secretion of sIgM into the extracellular milieu (4, 5). At the genetic and structural level, the murine B-1 cell compartment, recognized as a principal source of natural IgM antibodies, exhibits a recurrent and conserved immunoglobulin repertoire (6, 7).

Eumycota possesses an exceptional capacity to detect and adapt to dynamic environmental cues, enabling them to manipulate their physiology in response to fluctuating or extreme ecological milieu. Through this remarkable phenotypic plasticity, they engage in a spectrum of interactions with plants, protists, worms, insects, animals, and humans, ranging from mutualism and commensalism to latent colonization and overt pathogenesis. Notably, numerous fungal species, including both pathogens and benign commensals, have coevolved with mammalian hosts over evolutionary timescales spanning millions of years (8, 9). This prolonged association has driven the emergence of sophisticated immune escape mechanisms, whereby fungi exploit complex cellular and molecular strategies to subvert host defenses and establish successful colonization (10–14). In the setting of life-threatening fungal infections such as those caused by *Candida* spp., *Aspergillus* spp., and *Cryptococcus* spp., the structural properties of IgM, particularly its pentameric configuration and multivalent binding capacity, enable potent interactions with the highly repetitive carbohydrate and mannoprotein epitopes abundant on fungal cell walls. Investigations into host immune recognition of fungal polysaccharides in severe mycoses have revealed a consistent immunological pattern. Conserved polysaccharide components integral to the cell walls of various pathogenic fungi such as β-glucans, mannans, exopolysaccharide capsules, chitin, and chitosan are among the most potent activators of host immune responses and are recognized as Pathogen-Associated Molecular Patterns (PAMPs), largely through engagement with soluble and membrane-bound Pattern Recognition Receptors (PRRs), including dectin-1, mannose receptors, and Toll-like receptors (15, 16). These interactions initiate pro-inflammatory signaling cascades and contribute to fungal clearance. However, pathogenic fungi have evolved unique exopolysaccharides that act as protective masks, including complex capsules and secreted glycoproteins or metabolites, that effectively obscure these immunogenic motifs from host surveillance (17–19). These unique structural adaptations not only shield conserved glycans from immune surveillance but may also actively manipulate or suppress host inflammatory processes, thereby facilitating immune escape and persistent infection in susceptible individuals (20, 21).

IgM facilitates effective recognition and elimination of fungal elements, even amid antigenic variability and morphological shifts or dimorphism characteristic of these opportunistic pathogens. Additionally, IgM plays a crucial role in initiating the classical complement pathway, thereby promoting opsonization, enhancing phagocytic clearance, participating in opsonophagocytosis, and contributing to the early containment of fungal proliferation and filamentation, particularly in the case of filamentous fungi (22). Despite these mechanistic advantages, the immunoprotective and therapeutic roles of IgM in systemic mycoses remain underexplored. This knowledge gap is critical given the escalating global burden of invasive fungal diseases, particularly in populations with weakened immunity where fungal dissemination is frequently observed (23, 24).

This comprehensive review integrates emerging evidence on IgM molecular basics and immunological response dynamics, with a focused discussion on its potential contributions to innate and acquired immunity against pathogenic fungi. The review will describe the molecular architecture of IgM, biological dynamics of B lymphocyte and the synthesis of IgM, protective and regulatory roles of IgM in anti-fungal defense, structural and functional convergences between IgM and lectins in glycans recognition, and investigations of the role of IgM binding against conserved fungal cell wall components. Finally, we will discuss future perspectives in IgM**’**s role in responses to and prevention of mycoses.

## 2 Molecular architecture of IgM

**Figure 1:**
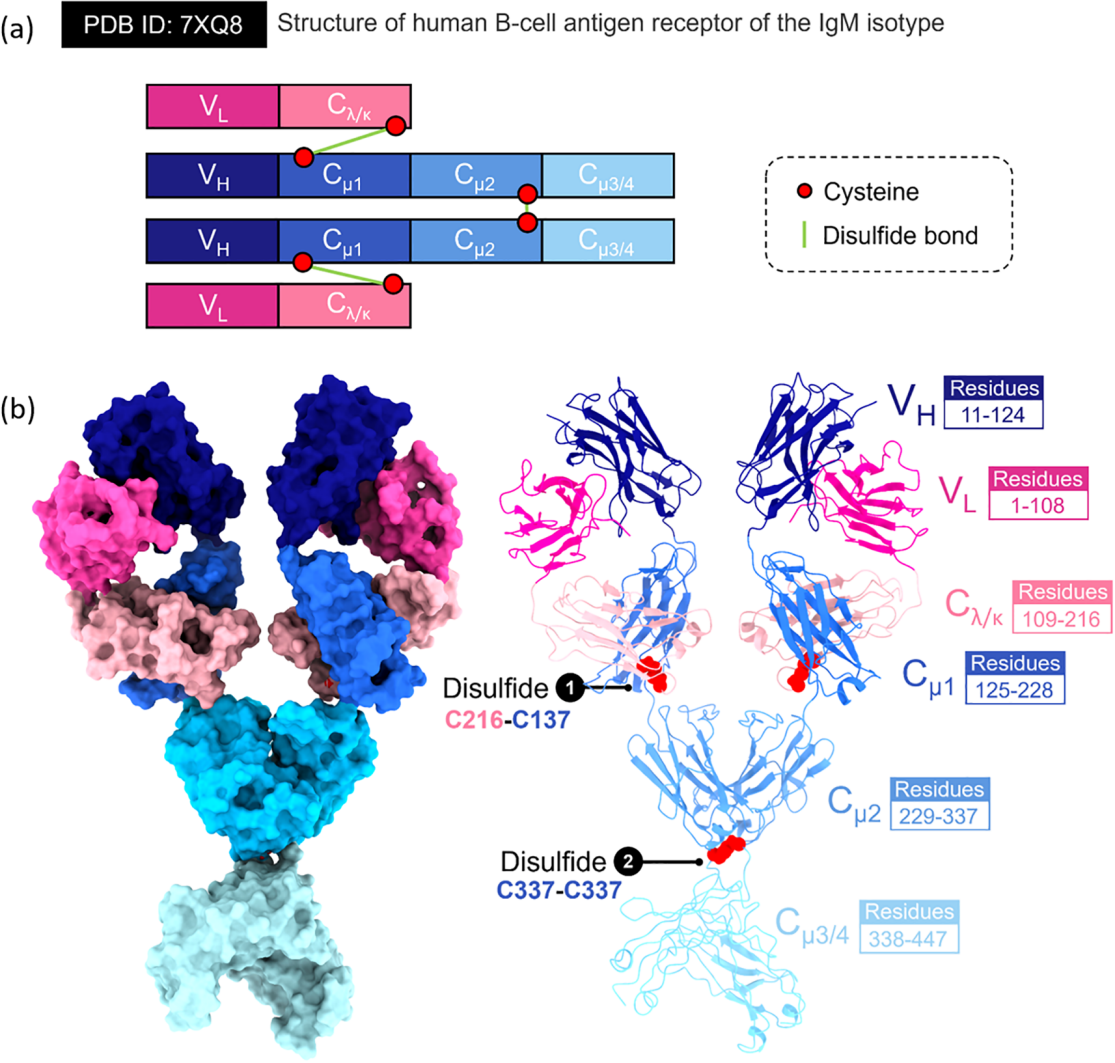
Structural features of the human B-cell antigen receptor (BCR) of the IgM isotype. This figure presents the molecular arrangement and interdomain architecture of the membrane-bound IgM-BCR (PDB ID 7XQ8), highlighting domain composition, disulfide connectivity, and domain-specific residue mapping. **(A)** Schematic representation of the IgM-BCR heavy and light chains. Each receptor comprises two identical heavy chains (VH–Cμ1–Cμ2–Cμ3/4) and two light chains (VL–Cλ/κ). Key cysteine residues (red circles) are involved in forming stabilizing disulfide bonds (green lines), including: (1) an interchain bond between Cys216 (in Cλ/κ) and Cys137 (in Cμ1), connecting heavy and light chains, and (2) a symmetric Cys337–Cys337 linkage between the Cμ2 domains of the two heavy chains. **(B)** left panel. Surface-rendered model of the IgM-BCR extracellular portion, showing spatial configuration and domain contacts. Individual domains are color-coded: variable light (VL, magenta), variable heavy (VH, dark blue), constant light (Cλ/κ, light pink), and constant heavy domains Cμ1 (blue), Cμ2 (cyan), and Cμ3/4 (light cyan). **(b)** right panel. Ribbon diagram of the full IgM-BCR, emphasizing domain boundaries and structural connectivity. The corresponding amino acid residues are indicated for each region: VL (residues 1–108), VH (11–124), Cλ/κ (109–216), Cμ1 (125–228), Cμ2 (229–337), and Cμ3/4 (338–447). Disulfide bridges are highlighted in red, with annotations identifying cysteine pairings essential for BCR assembly and structural integrity.

IgM is recognized as the prototype antibody of the early-phase or primary immune response, characterized by a distinctive and very complex molecular architecture. Over the past century, conceptual understanding of IgM has undergone substantial revision. Initially believed to adopt a rigid, symmetrical oligomeric form, IgM is now understood to be a structurally diverse and dynamic assembly, a feature that underlies its multifaceted immunological functions. The fundamental subunit of IgM or monomeric IgM is the Cµ_2_L_2_ structure, comprising two heavy (µ) chains and two light chains (κ or λ). Each heavy chain terminates in a carboxyl tailpiece of 18 amino acids (PTLYNVSLVMSDTAGTCY), which plays a crucial role in inter-protomer disulfide bonding, enabling the formation of higher-order polymeric structures (2, 25). These protomers or monomers assemble into pentamers, typically stabilized by a single joining (J) chain, or into symmetric hexamers in the absence of the J-chain (**Figures 1A, B**) (26). IgM possesses an approximate molecular weight of 900 kDa in its pentameric form and 1,050 kDa as a hexamer (2).

Early work using negative-stain electron microscopy and low-resolution X-ray scattering studies portrayed pentameric IgM as a planar, star-shaped structure, while hexameric IgM was viewed as a closed symmetric ring. Although the functional significance of the hexameric IgM configuration remains incompletely elucidated, its emergence is hypothesized to result from structural aberrations in the μ heavy chain or the absence/dysregulation of the J-chain within the canonical pentameric form (27). These structures were thought to be stabilized by β-sheet stacking interactions among the tailpieces of the µ-chains (28). The J-chain was historically considered as a structural appendage with a minor role in symmetry and function. Furthermore, the antigen-binding Fab fragments were assumed to adopt fixed, equivalent orientations to support multivalent antigen engagement and allow simultaneous C1q binding for complement activation (29).

**Figure 2:**
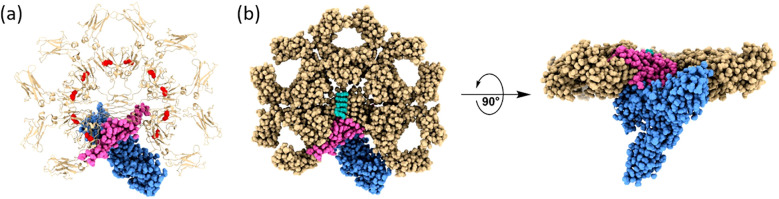
Structural architecture of the human IgM-Fc pentamer in complex with the polymeric immunoglobulin receptor (pIgR). Structural representation of the human secretory IgM (sIgM) pentamer in complex with the ectodomain of the human polymeric immunoglobulin receptor (pIgR), resolved by cryo-EM (PDB ID 6KXS). **(a)**. Demonstrates a transparent ribbon model, highlighting the 5 Cμ regions (golden brown), the joining (J) chain (magenta), and the pIgR ectodomain (blue); the disulfide bonds on the Cμ3 are highlighted in red. **(b)** left panel. Exhibits a top-down surface rendering of the complex, demonstrating the central pentameric symmetry of IgM and the incorporation of the J chain, which serves as a nucleating element and facilitates pIgR binding. **(b)** right panel. Exhibits a side view rotated by 90°, highlighting the vertical alignment of the Fcμ-J-pIgR interface and the central positioning of the J chain and pIgR within the IgM pentamer. This architecture forms the basis of the molecular mechanism by which IgM engages pIgR for epithelial transcytosis and is responsible for mucosal immunity.

During the COVID-19 crisis, a transformative shift occurred between 2022 and 2023 with the advent of high-resolution single-particle cryo-EM being applied to study IgM, which revealed that previous models were oversimplified or inaccurate. High resolution single - particle cryo EM studies of full length human IgM pentamers revealed a rigid Fc core (Cμ3–Cμ4–tailpiece–J chain) with considerable flexibility in the Fab-bearing Cμ2–Cμ1 subdomains (30, 31). These structures showed clear asymmetrical structure. In pentameric IgM, the J-chain inserts asymmetrically into the Fc core, inducing local distortions in adjacent protomers and disrupting the presumed fivefold symmetry. Additionally, the Cμ3–Cμ4 domains of the µ-chains were found to form an amyloid-like core stabilized by cooperative β-sheet interactions with both the tailpiece and J-chain. In contrast, the Cμ1–Cμ2 region, which accommodates the Fab fragments, exhibited a highly flexible hinge-like architecture, attributable to a tunable interface between Cμ2 and Cμ3, rather than the canonical hinge seen in IgG. This revised structural framework helps explain longstanding functional observations (**Figures 2A, B**). For example, pentameric IgM binds specifically to the pIgR to mediate transcytosis across epithelial barriers. By comparison, the hexameric assembly facilitates more efficient activation of the complement cascade, especially under conditions of critically low antigenic density (32). Molecular dynamics simulations further support these findings, showing that Fab angles vary in response to external influences such as Fc glycosylation or Fc receptor engagement. Moreover, the functional characteristics of IgM antibodies are also uniquely defined by the structural composition of their μ constant region, which lacks the capacity to engage and activate Fcγ receptors, a property observed in certain IgG subclasses. Under physiological conditions, B-1 cell responses are typically restricted to class-switching toward IgG isotypes with minimal or absent Fcγ receptor-binding activity, such as murine IgG3 (33).

Currently, an integrative model has been proposed, positing that co-translational oxidation of the tailpiece, in concert with J-chain insertion, acts as a molecular decorator to direct IgM assembly toward the pentameric form. This pentamer is functionally regulated as certain Fab arms are spatially constrained to match the pIgR-binding site and facilitate efficient transcytosis. In contrast, in the absence of the J chain, such as in certain malignant B cells, IgM assembles into symmetric hexamers, exposing all six C1q binding sites simultaneously, thereby enhancing complement activation and cytolytic capacity. However, this configuration precludes pIgR binding and thus impairs mucosal transport. This new understanding emphasizes the importance of structural plasticity. The coexistence of a stable, amyloid-like core with localized asymmetry and rotational flexibility at the Cμ2–Cμ3 interface enables IgM to adapt to diverse immunological contexts, including microbial agglutination, cell surface receptor cross-linking, and fine-tuned immune signaling. These structural insights have catalyzed new directions in antibody engineering. Site-specific mutations within the tailpiece can bias IgM assembly toward either the pentameric or hexameric form, depending on the intended application such as enhancing mucosal delivery in vaccines or maximizing cytotoxicity in cancer immunotherapy. These advances will deepen our molecular understanding of immune mechanisms and facilitate the design of highly specific polymeric antibodies with tunable immunological functions tailored to clinical needs (34).

## 3 Biological dynamics of B lymphocyte and the synthesis of IgM

Naïve B lymphocytes are classically divided into 3 primary subsets including B-1 cells, follicular B cells, and marginal zone (MZ) B cells. B-1 cells, predominantly found in the peritoneal and pleural cavities, are further subdivided into B-1a and B-1b cells, with distinct phenotypic and functional properties (35). Together with MZ B cells located in the splenic marginal zone, B-1 cells constitute a specialized, pre-activated B cell compartment adept at mounting rapid responses to T cell–independent (TI) antigens. These cells are enriched in polyreactive B cell receptors (BCRs) with low affinity but broad specificity, encoded by unmutated germline immunoglobulin gene segments. Their repertoire is evolutionarily skewed to recognize conserved microbial motifs, including fungal polysaccharides, lipopolysaccharides, and repetitive carbohydrate-associated epitopes. B-1 cells arise from fetal liver precursors and self-renew in peripheral niches, whereas MZ B cells differentiate from transitional stage 2 B cells under the influence of Notch2 signaling and B cell-activating factor (BAFF)-mediated maturation.

Upon TI antigen recognition, particularly those displaying multivalent structures, BCR crosslinking triggers signaling cascades (36). These involves activation of Src-family kinases Lyn and Fyn, phosphorylation of immunoreceptor tyrosine-based activation motifs (ITAMs) in Igα/Igβ, and subsequent recruitment of Syk. Concurrently, TI antigens derived from fungi such as yeast zymosan (β-glucans), mannan, phospholipomannan, glucuronoxylomannan (GXM), and galactomannan activate pattern recognition receptors, particularly Toll-like receptors (TLRs) such as TLR2 and TLR4. These inputs integrate with BCR signals via MyD88-dependent pathways to augment transcriptional programs mediated by NF-κB and AP-1 (37). This synergy accelerates plasmablast differentiation and enhances IgM production in response to a wide array of conserved microbial ligands (38–40).

TI antigen–induced IgM responses are extrafollicular in nature and peak rapidly typically within 3–5 days without the need for germinal center (GC) formation (41). Differentiation into CD138^+^ IgM-secreting plasmablasts is driven by the transcriptional repressor Blimp-1, repression of Pax5, and induction of XBP1, which supports the unfolded protein response necessary for high-volume immunoglobulin synthesis (42). Secreted IgM, predominantly in a pentameric form, exhibits high avidity for repetitive antigens, enabling potent agglutination, neutralization, and activation of the classical complement pathway via C1q engagement. In contrast to IgG, IgM exhibits markedly greater potency in complement activation; a single antigen-bound IgM molecule is sufficient to initiate the complement cascade and induce lysis of red blood cells, whereas the equivalent effector function necessitates the engagement of thousands of IgG molecules (4).

Importantly, TI responses give rise to long-lived plasma cells that localize to bone marrow niches and contribute to a persistent pool of natural IgM antibodies. These antibodies provide constitutive basal defense against bloodstream and mucosal invading pathogens (43). Their production is tightly regulated by the IgM Fc receptor (FcμR), expressed on B cells and follicular dendritic cells, which serves to prevent excessive activation and potential autoreactivity (44). In addition to antimicrobial defense, natural IgM also contributes to immune homeostasis by facilitating the clearance of apoptotic debris and microbial translocation across mucosal barriers, thereby maintaining a balanced microbiota–immune system interface (45). Crystallographic analyses have elucidated the structural features governing antigen specificity of natural antibodies. Notably, anti-phosphorylcholine (PC) IgM antibodies, a hallmark of the B-1 cell repertoire, are largely encoded by the germline VH gene segment VHS107.1. This segment forms a recessed antigen-binding pocket tailored for PC binding, representing an evolutionarily conserved architecture for innate-like BCRs. Mice lacking this specific *VH* segment retain overall immunocompetence but exhibit impaired IgM responses to PC-containing antigens on apoptotic cells and pathogens (33, 46).

Beyond direct microbial neutralization, pentameric IgM plays a crucial role in bridging innate and acquired immunity. By forming immune complexes and depositing C3b via classical complement activation, IgM enhances antigen uptake by dendritic cells through complement receptors CR1/CR2 and FcμR (47). This promotes antigen presentation and T cell priming. Complement mediated opsonization of antigen also enhances its retention and display within the follicle, providing a foundation for GC recruitment, isotype switching, and affinity maturation. Consequently, IgM produced in early TI responses functions not only as a first-line defense but also as an adjuvant facilitating follicular B cell activation, T cell recruitment, and long-term humoral memory (48).

Although classically associated with innate immunity, IgM also plays a role in the early phases of acquired immune responses. Upon activation by cognate antigen, naïve follicular B cells migrate from the follicle, proliferate, and differentiate into short-lived IgM-secreting plasmablasts within secondary lymphoid tissues (45). Antigen presentation to CD4 T follicular helper (T_FH_) cells via MHC–TCR interactions induces B cell reentry into the follicle and GC formation, where somatic hypermutation and class switch recombination refine antigen affinity and isotype profile (49). This process generates long-lived plasma cells and memory B cells that contribute to sustained protective immunity (50–52).

Notably, a subset of IgM+ memory B cells harboring somatically mutated V regions has been identified, suggesting a post-GC origin and indicating that IgM is not merely an early response isotype, but a component of durable immunological memory (50, 52). Understanding the cellular and molecular underpinnings of TI IgM responses offers critical insights for the rational design of polysaccharide–protein conjugate vaccines. Such strategies aim to harness both T-independent and T-dependent pathways to elicit broad and long-lasting immunity across age groups and immunocompetence states. Particularly in medical mycology, it is widely recognized that fungal cell walls and their surface-derived components are highly conserved structures that effectively stimulate inflammation and may serve as candidates for stimulating pan-protective antibodies (53, 54).

Systemic and opportunistic fungal infections are closely associated with HIV infection, particularly in advanced HIV disease, and that HIV exerts a significant influence on the structural modification of antibodies, especially IgG, thereby affecting their binding affinity and neutralization ability against target epitopes. The VH3 gene, which belongs to the immunoglobulin heavy-chain variable region gene family, plays a central role in shaping the antigen-binding specificity and diversity of B cell receptors and antibodies. Previous studies examined VH3 gene expression in HIV-infected and uninfected children, including those with a history of invasive pneumococcal disease (IPD). The results found that IgG VH3 expression was significantly reduced in HIV positive children without IPD, while IgM VH3 expression inversely correlated with viral load, particularly in HIV^+^/IPD^+^ cases. In contrast, IgG VH3 expression positively correlated with viral load in these children. These findings suggest that HIV infection alters the B cell VH3 repertoire and may impair protective antibody responses, potentially increasing the risk of pneumococcal infections in some HIV^+^positive pediatric patients (55). Additionally, other studies have investigated the impact of HIV infection on VH3 gene expression in B cells, revealing a 45% reduction in VH3 family representation among serum IgM proteins in patients with advanced HIV disease. However, the proportions of VH3 within VH IgM and IgD mRNA from peripheral B cells remained comparable to those in healthy controls, indicating that the naïve B cell repertoire is preserved. Alterations were primarily observed in the memory B cell producing IgG population, where VH3 expression decreased and VH4 increased. This suggests that HIV predominantly affects VH gene usage in activated, rather than resting naïve B cells. The preservation of a normal VH3 repertoire in naïve B cells, particularly those contributing to IgM production, including genes encoding putative HIV gp120 binding sites supports the capacity to generate robust antibody-mediated responses. Thus, these findings indicate that naïve B cells retain normal VH3 expression and that the capacity for IgM-mediated antibody production remains intact despite HIV infection (56).

## 4 The protective and regulatory roles of IgM in anti-fungal defense

The immunological role of IgM in infections caused by clinically significant fungi has not been as prominently recognized as that of other immunoglobulin isotypes, such as IgG, IgA, and IgE (59–61). However, work on *Pneumocystis murina* and *Cryptococcus neoformans* highlight the profound importance of IgM in response to invasive mycoses. Natural IgM antibodies exhibit broad specificity, enabling recognition of diverse microbial determinants, including viral antigens and bacterial toxins (57). This extensive cross-reactivity suggests a critical role for natural IgM in providing frontline protection against previously unencountered pathogens. Owing to the high avidity conferred by their polymeric structure, natural IgM antibodies are thought to play a pivotal role in the early containment of microbial invasion, effectively bridging the temporal gap before the activation of a pathogen-specific acquired immune response (45).

A significant study conducted by Rapaka et al. demonstrated that natural IgM antibodies targeting conserved fungal cell wall glycans represent an evolutionarily conserved component of immunity. *P*. *murina* is an atypical host−specific fungal pathogen that colonizes alveolar spaces in mice, existing as both trophic forms (thin, pleomorphic cells that adhere to alveolar epithelial surfaces) and cysts (thick−walled spore−containing structures), which facilitate airborne transmission. Baseline serum levels of IgM specific for β−1,3−glucan and chitin are detectable in diverse species including BALB/c and C57BL/6 mice, germ−free mice, catfish, and primate cord blood, which reveals a germline−encoded specificity maintained across phylogeny and poised to detect conserved fungal polysaccharide motifs. Upon respiratory exposure to *P*. *murina*, the steady−state IgM concentrations of 200 to 600 ng/mL in mice increased within two days of intratracheal challenge and returned to baseline by day 7, enabling rapid containment of both trophic and cyst stages without engaging slower GC pathways. Passive transfer of wild−type serum into severe combined immunodeficiency (SCID) mice accelerated fungal clearance and elevated pulmonary IL−1**β** and IL−6, demonstrating that serum IgM is both necessary and sufficient for early disease control (58).

Natural IgM also orchestrated innate cellular responses against *P*. *murina*. For example, opsonization of *P*. *murina* trophic forms or zymosan particles (fungal-derived cell wall β−glucan preparation) with IgM enhanced migration of pulmonary dendritic cells to mediastinal lymph nodes and amplified their production of TNF, IL−1β, IL−6 and IL−12p40. Mice lacking secreted IgM exhibited delayed clearance of both cyst and trophic forms and diminished early cytokine responses, confirming the central importance of IgM−mediated opsonophagocytic and cytokine−driven mechanisms in antifungal immunity. Beyond its immediate effects, this study revealed that secreted IgM shapes acquired immunity. In secreted IgM−deficient mice, the populations of Th2 (IL−5) and Th17 (IL−17) cells in draining lymph nodes 14 days after infection were markedly reduced while IFN−γ responses remained intact. These mice also showed altered antibody class switching, with reduced anti−*P*. *murina* IgG1 and mucosal IgA, but increased IgG2c. Thus, early IgM responses play a dual role by exerting direct antifungal effects against both stages of *P*. *murina* and by manipulating later immune events, such as T helper cell polarization and B cell isotype switching, thereby linking innate sensing to acquired immunity. Apart from studies involving *P*. *murina*, a murine-specific fungal pathogen, the immunological role of IgM in infections caused by clinically significant fungi has not been as prominently recognized as that of other immunoglobulin isotypes, such as IgG, IgA, and IgE (59–61).

The research group led by Liise-anne Pirofski has systematically elucidated and brought to prominence the functional importance of IgM within the context of systemic fungal infections, particularly in the case of *Cryptococcus neoformans* (62). The early immune response to *C*. *neoformans*, appears to rely significantly on natural IgM antibodies, whose regulatory potential can be conceptually framed within the idiotypic network (IT) theory proposed by physiology or medicine Nobel Prize laureate Niels Kaj Jerne in 1974 (63, 64). This theory suggests that antibodies not only recognize external antigens but also bear unique internal antigenic determinants **“**idiotopes**”** that can be recognized by other antibodies, creating a dynamic and self-regulating immune network. Natural IgM, largely derived from B-1 cells and polyreactive, is ideally positioned to initiate this network due to its germline encoded specificity for conserved microbial structures. In this context, IgM (Ab1 or antigen-specific antibody) may recognize fungal cell wall–associated PAMPs and subsequently elicit anti-idiotypic responses (Ab2), which have the potential to modulate immune activation and tolerance. Supporting this framework, experimental evidence shows that mice deficient in sIgM exhibit significantly increased susceptibility to severe pulmonary cryptococcosis by promoting a shift toward a more robust and inflammatory immune response, demonstrating its essential, non-redundant protective function during the early phase of infection. Moreover, anti-idiotypic responses may mimic fungal epitopes and facilitate a form of immunological imprinting or memory, even prior to full activation of acquired immunity (65–68). The investigation into *C*. *neoformans* titan cell formation highlights a foundational role for natural human IgM in directly inhibiting fungal morphological transitions that enhance pathogenicity. Titan cells are enlarged, polyploidy fungal forms that resist immune clearance. *In vitro*, IgM, but not IgG, binds β-glucan structures on the fungal cell wall and inhibits titan-like cell formation, reduces capsule size, and alters cell wall structure. The transcriptional alteration of genes responsible for cell wall biosynthesis and stress adaptation (e.g., CHS, AGS1, FKS1, RIM101, HOG1) further supports a mechanism wherein IgM modulates fungal virulence by both structural and transcriptional reprogramming. These findings establish a non-opsonic, innate immunomodulatory role for natural IgM in early fungal control (69). Additionally, HIV-associated cryptococcal immune reconstitution inflammatory syndrome (IRIS) is a paradoxical hyperinflammatory condition that may arise following the initiation of antiretroviral therapy (ART**).** Baseline deficiencies in IgM specific to fungal antigens such as GXM and β-glucans, in contrast to IgG or IgA, are associated with an increased risk of IRIS developing. Notably, this IgM-specific deficit does not reflect B-cell depletion, but rather suggests a qualitative or functional impairment in the IgM response. Inadequate IgM activity may permit unchecked fungal antigen persistence, which in turn predisposes individuals to exaggerate immune activation during immune reconstitution. These findings highlight the role of natural and antigen-specific IgM not only as antifungal effectors but also as key modulators of immune homeostasis in HIV individuals (70).

The evidence points to the essential role of IgM memory B cells and IgM antibodies in antifungal defense, particularly in protecting against *C*. *neoformans*. IgM memory B cells are identified as both biomarkers and effectors; their reduced frequency in HIV individuals is strongly associated with past and future cryptococcosis. These cells contribute to host defense through T-cell-independent mechanisms, rapidly producing natural IgM antibodies that target fungal polysaccharide antigens like glucuronoxylomannan and β-glucans. This reduction likely explains the functional IgM deficiencies observed in serological profiles, leading to increased susceptibility to fungal proliferation and IRIS. Complementing this mechanistic insight, experimental evidence from animal models provides direct support for the protective role of IgM: a human monoclonal 2E9 IgM antibody, specific for GXM and derived from a vaccinated donor, significantly prolonged survival in a murine model of cryptococcosis. This protection was mediated through complement activation, highlighting the classical role of IgM in promoting opsonization and clearance via C3 deposition. The monoclonal antibody VH3 gene usage reflects patterns typical of natural anti-polysaccharide responses. Taken together, these findings support the concept that IgM memory B cells and IgM antibodies are not only predictive of disease risk but also critical for fungal control, offering a compelling rationale for their application in vaccine design and therapeutic antibody development (68, 71).

GXM-based cryptococcal polysaccharide antigen (CrAg) is widely recognized as a reliable marker for monitoring treatment response and predicting prognosis in disseminated cryptococcosis (72, 73). Recent analyses have revealed that total plasma IgM levels are generally elevated in HIV individuals with asymptomatic cryptococcal antigenemia compared to those without antigenemia, a relationship that remains significant after adjusting for age, sex, and CD4^+^ level. This observation suggests heightened B-cell activation within the CrAg positive population. However, when examining antigen-specific IgM responses, evidence indicates a reduction in IgM antibodies targeting cryptococcal GXM (GXM-IgM) among CrAg positive individuals, although these differences often do not reach statistical significance following multivariate adjustment. Similarly, IgM antibodies against laminarin, a β-glucan component of fungal cell walls, are also decreased and inversely correlated with CrAg levels. Collectively, these findings suggest that despite an overall increase in total IgM, the specific natural IgM responses implicated in protective immunity against *C*. *neoformans* may be diminished in individuals with antigenemia. This imbalance likely reflects an impaired functional humoral response, which may contribute to the heightened susceptibility to invasive cryptococcal meningitis observed in this high-risk population (74).

There are additional studies that demonstrated important functional roles of IgM in host defense against other clinically relevant fungi, including *Candida* spp., *Aspergillus* spp., and *Histoplasma capsulatum*. Even in the absence of prior exposure to fungal pathogens, a substantial fraction of serum antibodies in naïve mice exhibit reactivity against fungal antigens, including those derived from *Candida albicans*. This reactivity is largely attributed to natural antibodies IgM. Notably, passive administration of a monoclonal natural IgM antibody, 3B4, which recognizes both the self-antigen keratin and *C. albicans* germ tubes, was found to protect mice from *C. albicans*-induced mortality. The protective mechanisms of this antibody involve the suppression of germ tube formation and enhancement of phagocytosis through opsonization, thereby promoting macrophage-mediated fungal clearance (75, 76). These observations are supported by further murine studies demonstrating that administration of opsonizing antibodies confers protection against disseminated candidiasis.

The antigenic specificity of antibodies plays a critical role in determining their protective outcome. Han et al. demonstrated that β-1,2-mannotriose specific monoclonal antibodies, whether of the IgM or IgG3 isotype, enhanced resistance to disseminated and vaginal *C. albicans* infections in wild-type, SCID, and neutropenic mice (77–79). Structural studies indicated that recognition of β-1,2-mannotriose by IgG3 monoclonal antibodies is dominated by internal saccharide residues. Moreover, β-mannan-specific IgM monoclonal antibodies were shown to reduce the required dose of amphotericin B in murine infection models, supporting their utility in combination antifungal therapy (80, 81). An important observation that merits attention and discussion is that complement proteins are essential for the protective effects of *C. albicans* β-linked mannan specific IgM and IgG3 antibodies, as they enhance fungal uptake and killing by neutrophils (82, 83). Strengthening the case for IgM antibodies as therapeutic agents, the monoclonal antibody C7, which targets *C. albicans* cell wall proteins Als3p and enolase, demonstrated antifungal effects through three distinct mechanisms: direct fungal killing, inhibition of adhesion, and suppression of filamentation. Additional investigations revealed that the fungicidal effect of MAb C7 was linked to its disruption of iron uptake, a key element in the virulence of *C. albicans*. Importantly, MAb C7 also displayed cross-reactivity with other pathogenic fungi, including *C. neoformans*, *Scedosporium* (*Lomentospora*) *prolificans*, and *Aspergillus fumigatus*, suggesting broad-spectrum antifungal activity (84, 85).

Alliinase is a plant-based lyase mainly observed in garlic (*Allium sativum*) and other *Allium* related species. Functionally, its main role is to catalyze the conversion of alliin, a sulfur-containing amino acid, into allicin (diallyl-dithiosulfinate) when plant tissues are damaged (86). This reaction serves as a defense mechanism, producing allicin a reactive sulfur compound with broad-spectrum antimicrobial properties, including antifungal activity. An IgM monoclonal antibody targeting catalase B exhibited antifungal activity *in vitro*, while a novel conjugate of an *A. fumigatus*-specific IgM monoclonal antibody with alliinase significantly improved survival in immunosuppressed mice with pulmonary aspergillosis. This conjugate selectively targeted and killed *A. fumigatus* while avoiding damage to lung tissue (87, 88), demonstrating the potential of antibody-enzyme conjugation methods in antifungal therapy.

The outcome of infection by dimorphic fungi can also be modified by IgM antibodies. Passive immunization with IgM monoclonal antibodies specific to a histone H2B-like protein located on the cell surface of yeast phase of the thermally dimorphic fungus *Histoplasma capsulatum* significantly protected mice lethally challenged with *H. capsulatum* yeast cells. This protective effect was mediated through a complement receptor 3 dependent mechanism that altered the intracellular fate of the fungus within mouse J774.16 macrophages, along with increased levels of IL-4, IL-6, and IFN-γ in the lungs during both the early and late phases of infection. Similar outcomes were observed with protective IgG1 and IgG2a monoclonal antibodies against *H. capsulatum* HSP60, which prolonged survival in lethally infected mice (89–91).

While the interaction between the complement system and IgM is a topic of considerable interest, many facets of this relationship are not yet fully elucidated and warrant further study. The complement system is a key component of innate immunity, providing multifaceted protection against major fungal pathogens. A shared feature of encapsulated or thick-walled fungi such as *C. albicans*, *A. fumigatus*, and *C. neoformans* is their intrinsic ability to withstand direct lysis by the Membrane Attack Complex (MAC; C5b-9 complex). As a result, complement activity primarily shifts toward promoting opsonization to enhance phagocytic uptake and generating the potent chemoattractant C5a, which drives the recruitment and activation of phagocytes. The infections caused by *C. albicans* and *A. fumigatus*, the C5a–C5aR1 signaling axis is indispensable for mobilizing immune cells, while opsonization through receptors such as CR3 and CR4 contributes significantly to fungal clearance (22, 92). Notably, although it is not fungicidal, MAC deposition on *C. albicans* has been shown to modulate immune activity by boosting mitochondrial responses and enhancing phagocytosis (93, 94). Moreover, in the case of *C. neoformans*, antibody-driven protection further illustrates this alternative role, IgM depends on C3 for effective opsonization of the capsule, completely circumventing the requirement for MAC-mediated lysis (95). All together, these findings emphasize that complement contributes to antifungal immunity not by direct destruction but by labeling fungal pathogens for elimination and coordinating cellular immune responses.

Beyond the roles of C1q and the classical pathway, the contribution of soluble pattern recognition receptors (PRRs) in recognizing fungal glycans is also noteworthy. Soluble PRRs, including Mannose-Binding Lectin (MBL), which binds mannan and N-acetylglucosamine (NAG) on *A. fumigatus* and *C. albicans* (96) Ficolins, which recognize L-fucose, D-mannose, and N-acetylglucosamine on *A. fumigatus* (97–99) and pentraxins, which bind galactomannan on *A. fumigatus* and *Paracoccidioides brasiliensis* (100), operate alongside IgM, which are preconfigured to target conserved fungal glycans such as β-glucans and chitin. Both classes of molecules potently activate the complement system, with soluble PRRs primarily initiating the lectin pathway and IgM serving as the most potent trigger of the classical pathway. This convergence allows for the potential of both synergy and conflict. Synergy arises when the simultaneous binding of PRRs and IgM to a pathogen surface triggers parallel complement activation, leading to rapid and amplified C3b deposition and downstream effector functions, including the promotion of inflammation, phagocytosis, and leukocyte recruitment, thereby providing the host with complementary initiation platforms. Conflict may occur through steric hindrance effect, where binding of a large soluble PRR physically obstructs the epitope targeted by IgM, or through competition for shared ligands, where high concentrations of PRRs with strong avidity for repetitive glycan structures outcompete IgM for binding sites and potentially limit classical pathway activation and opsonization.

## 5 Structural and functional convergences between IgM and lectins in glycans recognition

Glycans, which are complex carbohydrates composed of monosaccharide units linked to other sugars, are integral components of all living cells. They occur both as free oligosaccharides and as covalent modifications of proteins (glycoproteins) and lipids (glycolipids), forming a diverse molecular landscape at the interface between host and microbe. Among these glycans are epitopes that can trigger potent immune responses, either as invariant microbial molecular patterns broadly conserved across taxa or as altered self-antigens emerging during apoptosis, infection, or malignant transformation. A notable subset of these epitopes originates from commensal microorganism, while others arise as neoantigens due to aberrant glycosylation associated with cellular stress or damage (101). The glycan-rich surface architecture of fungal pathogens is not static but dynamically regulated in response to environmental cues, including host immune pressure. Morphological transitions such as yeast-to-hyphae-pseudohyphae switching in *Candida* spp (102)., conidia-to-yeast transition in *Talaromyces marneffei* and *Paracoccidioides brasiliensis* (103, 104) or capsule enlargement in *C*. *neoformans* (105) are commonly associated with alterations in glycan composition and surface exposure. In addition, several fungi actively shed glycan components, such as GXM, galactoxylomannan (GalXM), or mannan residues, into the host extracellular milieu via extracellular vesicles (EVs), where they further manipulate host immunity (106–109). These mechanisms highlight the complex and dynamic functions of glycans, which serve not only as structural elements but also as critical modulators of host–pathogen interactions. From an immunological perspective, fungal glycans represent both a vulnerability and a weapon. While they offer conserved molecular patterns that can be targeted by innate immunity and protective antibodies, they are also exploited by fungi to evade detection, dampen inflammation, and facilitate chronic persistence. Consequently, glycans are increasingly recognized as promising targets for antifungal vaccine development and immunotherapy. For example, β-glucan-based conjugates and mannan-derived antigens are under investigation for their ability to induce protective T lymphocyte and antibody responses. Understanding the complex functions of specific glycan moieties in fungal virulence and host recognition is thus essential for advancing fungal immunology and designing effective therapeutic interventions (110).

Natural IgM exhibits a remarkable structural and functional resemblance to classical carbohydrate-binding lectins in their recognition of glycan motifs. This similarity arises from the inherent biochemical challenges posed by glycan antigens, which are composed of repeating monosaccharide units with variable linkage geometries, dense hydroxylation, and branched topologies. Unlike peptides or proteins, which form well-defined linear or conformational epitopes that engage deep, high-affinity antigen-binding deep pockets, glycans typically lack shape-complementary features required for such interactions. Instead, their complex structures necessitate a distinct mode of recognition that relies on shallow, solvent-accessible surfaces enriched with polar and aromatic residues that are an architectural hallmark characterized by a marked predominance of tryptophan and a shared enrichment of aromatic side chains found in both lectin CRDs and the variable regions of IgM (111–113). The variable regions of natural IgM antibodies, particularly those encoded by germline VH (µ) and VL gene segments with minimal somatic hypermutation, adopt conformations characterized by extended CDRs that provide an adaptable, relatively flat binding interface.

Structural analyses of glycan-reactive IgM clones, such as the murine IgM 3B4, reveal a predominance of aromatic and hydrophilic residues positioned to facilitate CH/π interactions and hydrogen bonding with sugar hydroxyl groups—mimicking the canonical binding strategies of lectins like DC-SIGN and mannose-binding lectin (MBL) (76, 114). Both IgM and lectins compensate for the inherently low affinity of individual protein-glycan interactions through multivalent binding. While lectins exhibit finely tuned molecular complementarity within their CRDs, they typically achieve dissociation constants (*K_d_*) in the micromolar to millimolar range, and their functional strength derives from multiple CBDs simultaneously engaging clustered oligosaccharide ligands on cell surfaces. When compared to antibodies, the *K_d_* for the binding of a single Fab domain to a monosaccharide unit ranges within the 100 nM to 100 μM, reflecting weak and readily reversible interactions. This limited affinity is inherently linked to the TI nature of most glycan antigens, which fail to induce germinal center reactions required for high-affinity B-cell selection and clonal expansion (115, 116). Similarly, natural IgM assembles into pentameric or hexameric structures with 10–12 antigen-binding sites in structurally adaptable conformations that promote increased avidity by facilitating simultaneous interactions with densely arrayed glycan epitopes. Moreover, the constant regions (Fc) stabilize the paratope (antigen-binding site; Fab) structure, maintaining its conformation for effective antigen binding. This structural support enhances the precision of glycan recognition, ensuring selective interaction with specific fungal epitopes, despite the typically low intrinsic affinity of individual Fab–glycan interactions (117, 118). This structural and functional convergence has profound immunological implications. Natural IgM functions as an innate-like immunoglobulin with pattern recognition capacity, recognizing conserved microbial glycans (β-glucans, mannan residues, and chitin) without prior antigen exposure.

Crystallographic studies and computational modeling continue to reveal the mechanistic parallels between IgM and lectins. Structural models of antibody Fabs bound to glycan epitopes exhibit binding surfaces remarkably similar in geometry and residue composition to the CRDs of lectins such as MBL (PDB ID: 1HUP**).** Both structures display an emphasis on surface-exposed polar and aromatic side chains that mediate interactions across the broad and shallow plane of glycan epitopes (119). These findings support a model in which convergent structural evolution has equipped natural IgM with the molecular toolkit necessary to recognize fungal glycans in a manner analogous to prototypical lectins. Overall, the structural similarity between glycan-specific natural IgM and lectins reflects a convergence of functional demands imposed by carbohydrate antigen recognition. By leveraging structurally pliable, low-affinity binding domains arranged in a multivalent configuration, natural IgM emulates the molecular design principles of lectins, thereby enabling effective recognition of conserved glycan motifs present on fungal pathogens. This convergence underscores the integral role of IgM in innate immune surveillance and highlights its capacity to function as a molecular lectin within the immunoglobulin repertoire.

## 6 Investigating the role of IgM binding against conserved fungal cell wall components: the evidence derived from preliminary computational approaches

Although traditionally underappreciated in comparison to protein or peptide-based antigens, glycans are now recognized as critical immunological targets. Their non-peptide, three-dimensional structures are often conserved across phylogenetically distant organisms resulting in their ability to function as universal molecular patterns that transcend species boundaries (120). This structural conservation facilitates robust cross-reactive immune recognition and places glycans at the center of both pathogen detection and immune manipulation. Glycan recognition is mediated primarily by IgM, which is a germline-encoded and evolutionarily conserved component of the mammal humoral immune system. In general, based on molecular architecture, IgM is especially well suited for glycan recognition. The structural features of IgM enables their ability to engage repetitive, weakly interactive glycan determinants with sufficient strength to support early immune containment and surveillance. Furthermore, the ability of IgM to recognize conserved glycan motifs across taxonomic groups suggests a role in broad-spectrum immunity and positions IgM as a functional link between innate and acquired immune responses (45, 121–123). Glycan antigens, especially those derived from pathogenic fungi, present a unique structural and immunological challenge. Unlike the linear or looped backbones of peptide epitopes, fungal glycans such as laminarin (β-1,3-glucan) and chitin exhibit extended, branched, and topologically complex architectures. These structures are composed of repeating monosaccharide units with variable linkage geometries. Such structural configurations make them inaccessible to the deep, shape-complementary paratopes typically evolved to recognize peptides and instead require a distinct mode of antibody engagement, exemplified by the variable regions (Vμ/VL) of IgM (124–127).

To perform preliminary *in silico* simulations, we designed the Fab domain of the monoclonal antibody CC5 (MAbCC5) based on its amino acid sequence, as reported by Figueiredo et al. (128). This murine IgM targets the chitotriose of *C. neoformans*. The designed Fab domain was modeled for its interaction with several well-established, conserved fungal cell wall components, which are conserved in most pathogenic fungi including chitotriose (chitin), laminarin, galactoxylomannan (GalXM), and eumelanin. The polyglycine was included as a negative control, consistent with its function in the previously reported *in vitro* assays (128).

**Figure 3:**
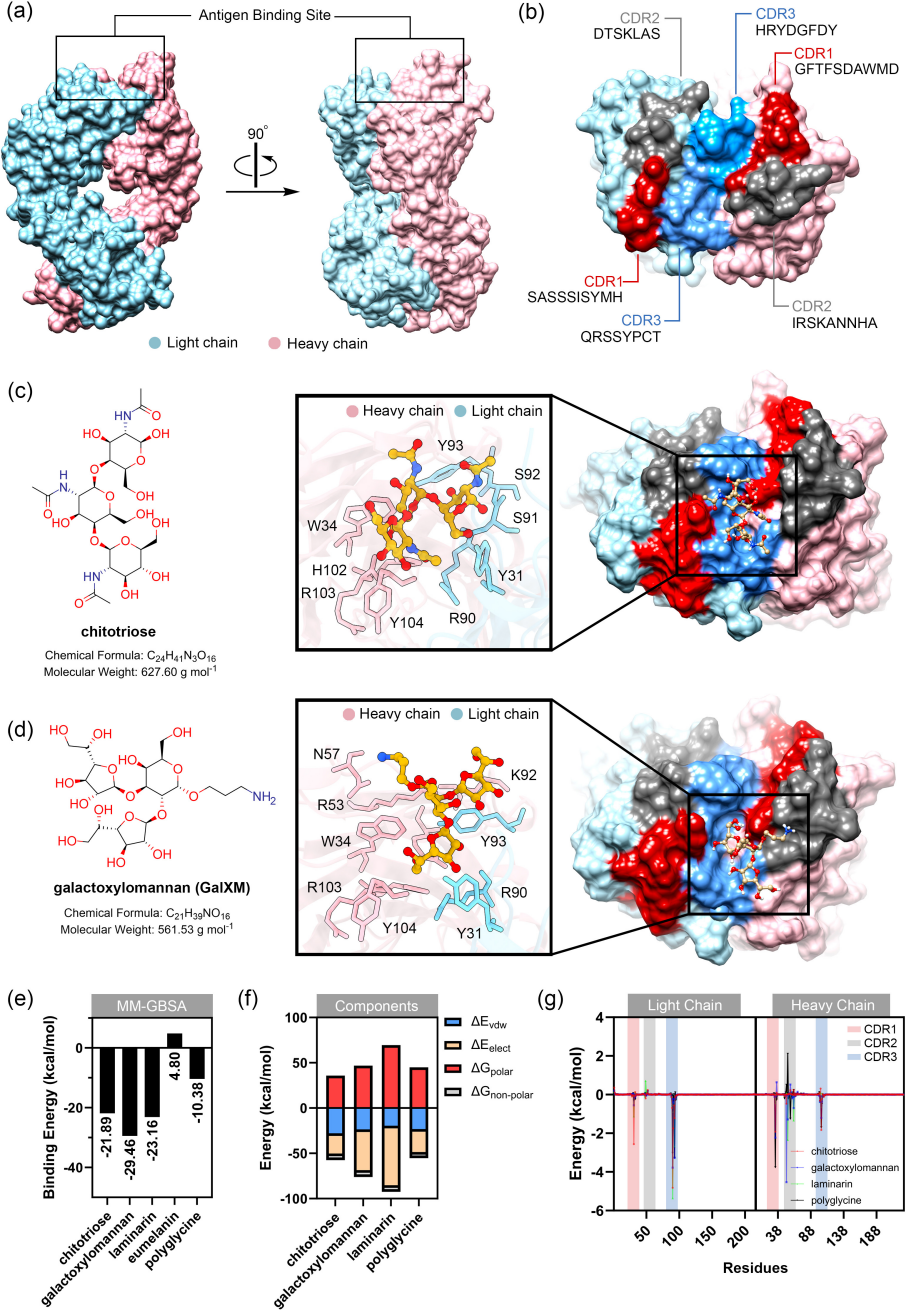
Preliminary study of binding between modeled MAbCC5 and selected glycans typically found in fungal cell wall. **(a)** Predicted structure of fragment antigen-binding region (Fab region). Antigen binding site is located at the top of the structure. Surface representation of the IgM Fab fragment highlighting the light chain (blue) and heavy chain (pink). Two orientations are shown: front view (left) and side view (right), with the antigen-binding site indicated. **(b)** Surface of antigen binding site. The structures are highlighted by red, gray, and blue for CDR1, CDR2, and CDR3, respectively. **(c)** Molecular structure of chitotriose (left) and its binding interface with IgM. The magnified-in inset shows hydrogen bonding and hydrophobic interactions between chitotriose and residues in both heavy and light chains. Key residues involved in binding are labeled, including W34, H102, Y93, and R90. **(d)** Molecular structure of galactoxylomannan (left) and its interaction with the IgM binding pocket. A close-up view highlights interactions with CDR residues including N57, R53, and K92. Glycan contacts span across the heavy and light chains. **(e)** Relative binding free energy calculated from MM-GBSA calculation. Binding to galactoxylomannan exhibits the strongest interaction energy. **(f)** Energy components of each complex. Decomposition of the total binding free energy into energetic components: van der Waals (ΔE_vdW), electrostatics (ΔE_ele), polar solvation (ΔG_polar), and nonpolar solvation (ΔG_nonpolar). **(g)** Per-residue energy decomposition analysis. Amino acid residues within CDR1, CDR2, and CDR3 that significantly contribute to binding are highlighted, supporting their central role in glycan recognition.

To gain insight into the interaction between MAbCC5 and fungal cell wall glycans, we subjected the structure of the Fab region of MAbCC5 to computational modeling. The crystal structure of a mouse B cell receptor (PDB ID: 8EMA) was used as a template for structure prediction. The amino acid sequences of the complementarity-determining regions (CDRs) of MAbCC5 were modeled according to a previous report (128). The CDRs of the light chain (GenBank: AAA63380.1) and heavy chain (GenBank: AMN90557.1), which share 96**%** and 81**%** sequence identity with MAbCC5, respectively, were aligned to the B cell receptor sequence using Clustal Omega (**Supplementary Figure 1**) (129). The full sequence used for structure prediction is provided in supplementary information (**Supplementary Table 1**). The complete Fab structure was predicted using AlphaFold 3 (130), as shown in **Figures 3A and B**. The resulting model displayed a quaternary structure similar to that of another carbohydrate-binding antibody (PDB ID: 1F4Y) (131), which is an antibody to a *Vibrio cholerae* O1 carbohydrate. However, analysis of the antigen-binding site for MAbCC5 revealed that the glycan-binding cavity is relatively small, and shallow compared to other antibodies, particularly IgG, which may account for the lower binding affinity observed.

**Figure 4:**
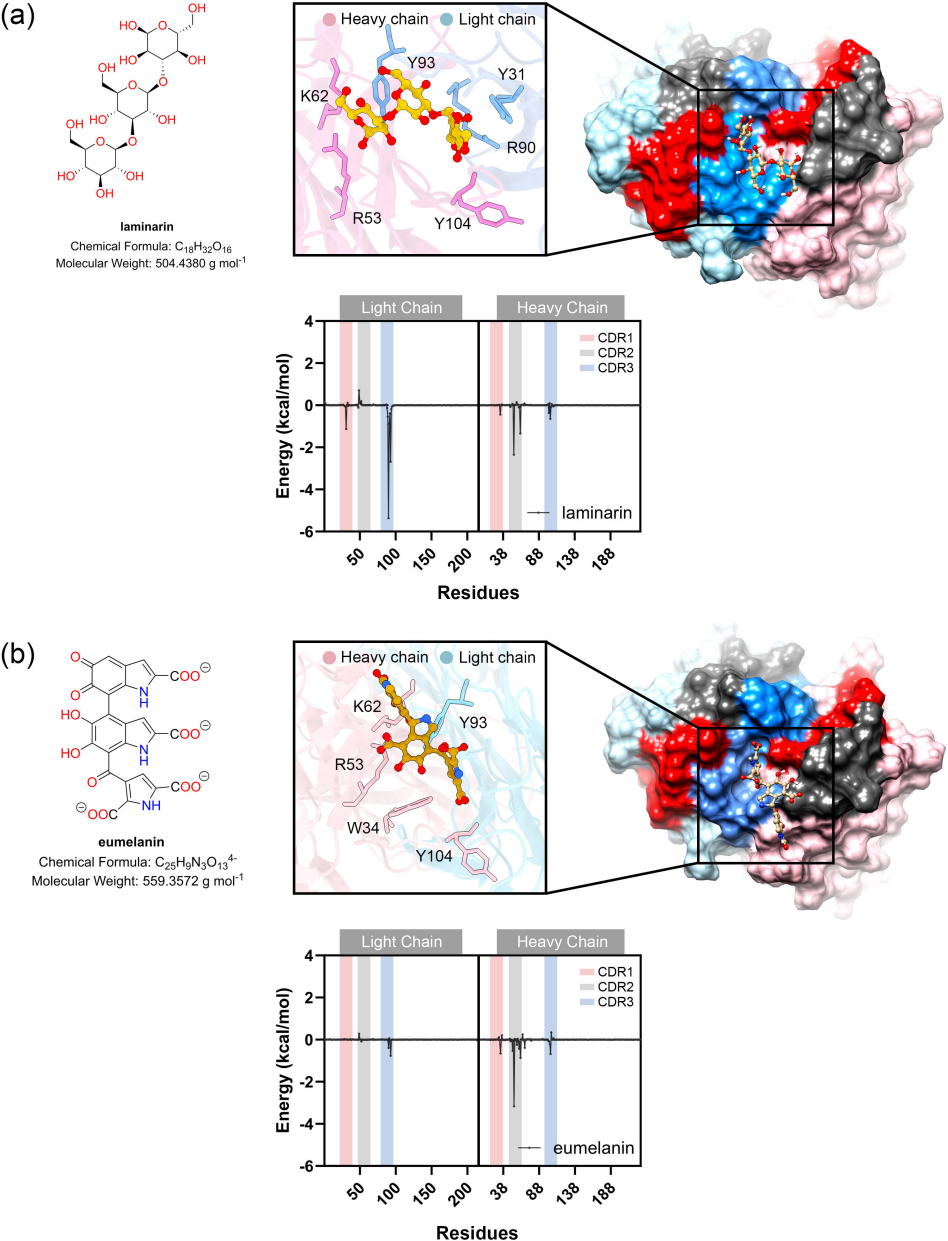
Molecular docking and MM-GBSA calculations of laminarin **(A)** and eumelain **(B)**. Molecular docking study of ligand binding pose within antigen binding site of modeled MAbCC5. The box shows the residues with contribution of binding energy less than -0.5 kcal/mol. Per-residue energy decomposition analysis using MM-GBSA calculation is shown in the diagram below.

The binding of fungal cell wall glycans, chitotriose, galactoxylomannan (GalXM), and laminarin, as well as eumelanin and polyglycine (with molecular weights comparable to those glycans) was predicted using the online tool CB-Dock2 (132), with the anti-carbohydrate antibody structure (PDB ID: 1F4Y) serving as the docking template. The results indicated that these ligands could be accommodated within the antigen-binding site, located in the cleft between the heavy and light chains. Relative binding free energy was calculated using the MM-GBSA method. The molecular mechanics calculations with generalized Born and surface area (MM-GBSA) calculation is one of the powerful technique for calculating the binding energy between carbohydrate and protein. This technique employs the molecular mechanics (MM) calculation for estimating van der Waals and electrostatic interactions in gas phase, along with the Generalized Born (GB) and surface area (SA) calculations for modeling the interaction in solvent phase (133). Among the tested ligands, GalXM exhibited the highest predicted binding affinity to the modeled MAbCC5 (–29.46 kcal/mol), followed by laminarin (–23.16 kcal/mol) and chitotriose (–21.89 kcal/mol) (**Figures 3C, D**, **4A** **).** In contrast, polyglycine, which served as a negative control *in vitro* (128), showed a substantially weaker binding energy of –10.38 kcal/mol. In contrast, eumelanin exhibited a positive binding energy, suggesting an unfavorable interaction likely driven by electrostatic repulsion between its negatively charged carboxylate groups and the CDR surface (**Figure 4B** **).**

Analysis of the energy components (**Figures 3E, F**, **4A, B**) revealed that binding to GalXM and laminarin was predominantly driven by electrostatic interactions, particularly hydrogen bonding. In contrast, chitotriose binding was primarily mediated by Van der Waals forces rather than electrostatic forces. This difference may be attributed to the higher abundance of hydroxyl groups in GalXM and laminarin, promoting stronger hydrogen bonding, whereas chitotriose contains fewer hydroxyl groups, with some replaced by amide functionalities. The presence of acetylaminyl groups in chitotriose appears to enhance non-polar interactions, thereby increasing Van der Waals interactions.

**Figure 5:**
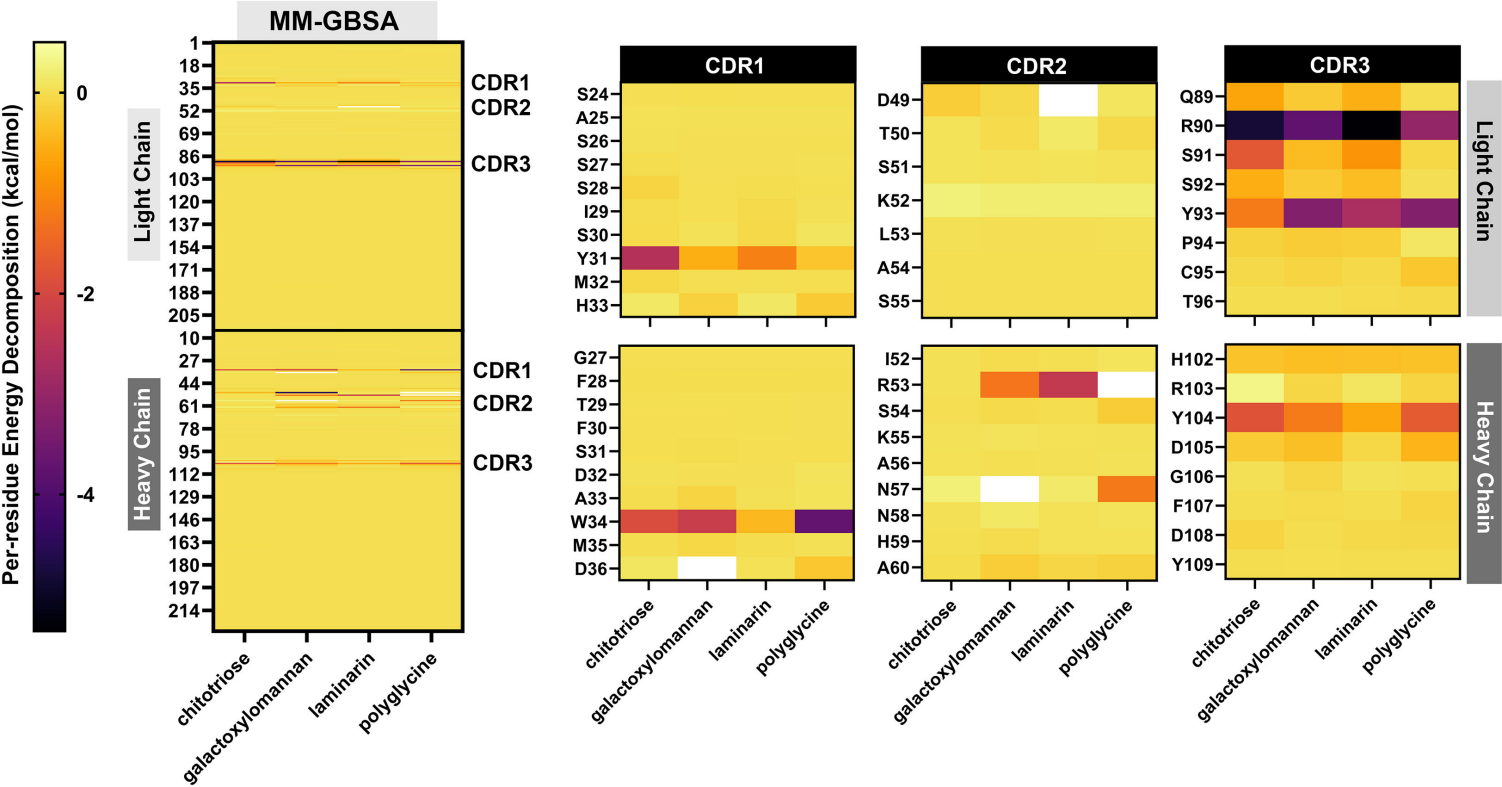
Demonstration by heat maps derived from per-residue energy decomposition analysis using MM-GBSA (Molecular Mechanics-Generalized Born Surface Area) calculations. The left panel shows a comprehensive overview of the energy contributions of individual residues in the modeled MAbCC5 antibody complex interacting with four ligands: chitotriose, galactoxylomannan, laminarin, and polyglycine. The heat map uses a color gradient from yellow (less favorable or neutral interaction) to red and purple (more favorable interaction, indicating stronger binding contributions), highlighting specific residues in both the light and heavy chains that significantly contribute to the binding energy. The right panel magnifies into the 3 CDRs of the light and heavy chains: CDR1, CDR2, and CDR3. These CDRs are critical for epitope recognition and binding specificity. The heat maps indicate which residues within each CDR are energetically important for binding each ligand, revealing variable binding patterns. For instance, residues Y31 in CDR1 (light chain) and W34 in CDR1 (heavy chain) show notable energetic contributions, suggesting key roles in ligand interaction. Differences in per-residue binding energies across ligands reflect distinct interaction modes, likely influenced by the chemical and structural properties of the glycan epitopes versus polyglycine.

Per-residue energy decomposition analysis (**Figures 3G**, **5**) indicated that all three glycans interacted extensively across the CDRs of the modeled MAbCC5, with notable contributions from the light chain CDR3. Key residues involved in glycan recognition included Y31, Q89, R90, S91, and Y93 of the light chain, and W34, R53, H102, and Y104 of the heavy chain. Many of these residues are aromatic, such as tyrosine, tryptophan, and histidine, which have previously been implicated in CH–π interactions known to enhance glycan binding (111). Notably, chitotriose predominantly interacted with the light chain residue R90 through hydrogen bonding, utilizing its hydroxyl and carbonyl groups. Similarly, GalXM and laminarin primarily engaged with the guanidinium and carbonyl groups of light chain residue R90 and heavy chain residue R51 via their hydroxyl moieties. These findings indicate that tyrosine and arginine residues are key contributors to glycan–antibody interactions. Significantly, R90 of the light chain and R53 of the heavy chain appear to play critical roles in glycan recognition, exhibiting stronger binding affinity to glycans compared to polyglycine.

**Figure 6:**
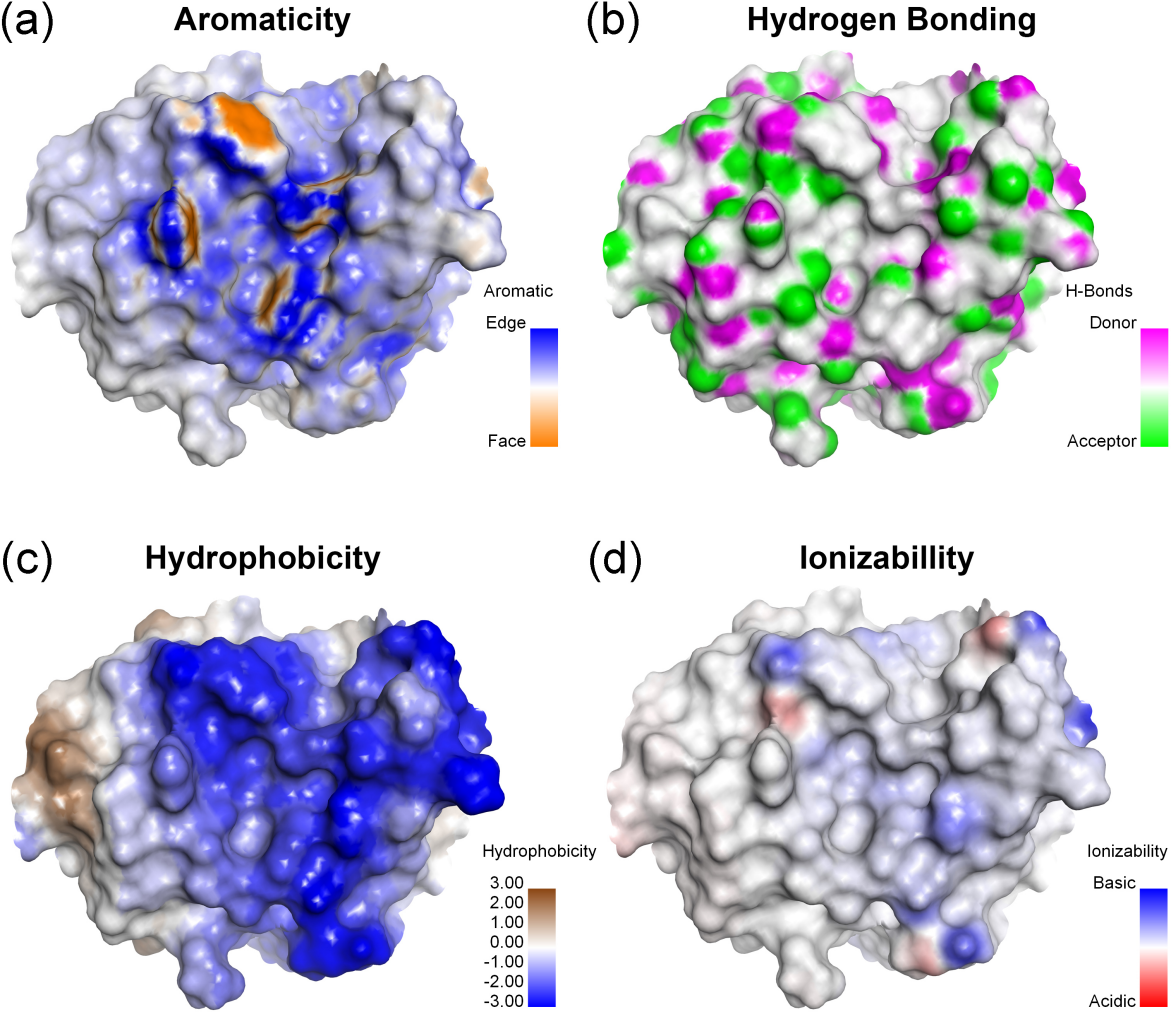
Surface representation of antigen binding site (Fab) of modeled MAbCC5. Surface property maps of a protein structure highlighting key biochemical characteristics relevant to molecular interactions. **(A)** Aromaticity: The distribution of aromatic residues is shown, with color mapping distinguishing between edge (blue) and face (orange) orientations of aromatic rings, relevant to CH–π or π–π interactions. **(B)** Potential for hydrogen bonding formation: Surface atoms capable of hydrogen bonding are identified, with hydrogen bond donors shown in magenta and acceptors in green, indicating potential interaction sites. **(C)** Hydrophobicity: The surface is colored based on hydrophobic potential, with blue regions indicating high hydrophilicity and brown regions indicating hydrophobicity, useful for identifying ligand-binding pockets. **(D)** Ionizability: Surface ionizable regions are visualized, with basic residues shown in blue and acidic residues in red, reflecting the electrostatic potential and protonation tendencies under the physiological pH.

Analysis of the contribution of aromatic structures at the antigen binding site (**Figure 6A**) revealed that the cavity contributed to strong aromatic contribution, especially the edge of the aromatic ring of residues Y31, W34, Y93, H102 and Y104. The presence of these residues allowed for π-interactions. Additionally, the presence of polar amino acids with hydroxyl, indole, and imidazole moieties enabling reactions with substances displaying high polarity, such as glycan or sugar moieties, through hydrogen bonding (**Figure 6B** **).** In the case of hydrophobicity, most environments of antigen binding site were enhanced by the strong hydrophilicity of the polar amino acids, especially tyrosine, aspartic acid, lysine, arginine, tryptophan and serine (**Figure 6C**). The binding site was constructed by light chain residues Y31, D49, K52, R90, S91, S92, Y93, and C95 and heavy chain residues W34, R51, R53, H102, R103, Y104, and D105. These residues contributed to the strong hydrophilicity of the binding site modeled with MAbCC5. The ionizability was effected by lysine and arginine for basicity and aspartic acid for acidity (**Figure 6D**). Most antigen binding sites showed a slightly positive charge caused by basic amino acids such as K52 and R90 of the light chain and R51 and R53 of the heavy chain.

These findings suggest that removal of the acetyl groups (-COCH_3_) of chitotriose to produce chitosan, which is a conserved component of the fungal cell wall found in nearly all pathogenic fungi, would result in a derivative that would lose its ability to bind the modeled MAbCC5. Under physiological conditions, the amine groups of chitosan are protonated and carry a positive charge. An analysis of the amino acid residues within the binding site revealed several regions with positive charge, including R51, K52, and R90. This electrostatic similarity would likely result in charge–charge repulsion, thereby making binding energetically unfavorable. Overall, the CDRs of mouse IgM anti- chitotriose of *C*. *neoformans* are enriched in polar and aromatic residues that form shallow, adaptable binding grooves. These grooves accommodate carbohydrate epitopes through a combination of hydrogen bonding, van der Waals forces, and CH–π interactions. Aromatic residues, such as tyrosine and tryptophan, facilitate planar stacking with sugar rings, while the guanidinium group of arginine can form multiple directional hydrogen bonds with hydroxyl groups on glycans. Electrostatic and entropic contributions, including the release of ordered water molecules upon binding, further stabilize these interactions despite the inherently low monovalent affinities. Moreover, this reactivity provides compelling evidence that the recognition pattern of the modeled IgM CC5 is highly conserved toward glycan motifs present in carbohydrate antigens and their derivatives. In contrast, its reactivity toward polypeptides and their derivatives, such as eumelanin and polyglycine, is relatively limited.

The nature of the IgM response to fungal melanin is a noteworthy topic that warrants rigorous academic investigation. We initially posited that the modeled MAbCC5 would exhibit significant binding to eumelanin. However, preliminary molecular docking results unexpectedly revealed an unfavorable binding interaction. This finding suggests that the Fab region of IgM maintains a considerable degree of structural conservation, thereby limiting promiscuous reactivity and enforcing selectivity for specific molecular configurations. It is important to consider this result in the context that the predominant isotype elicited against fungal melanin is IgM, which has been empirically demonstrated to be protective in the murine models (134–136). Therefore, there is strong rationale to pursue more refined studies to elucidate the precise molecular interactions between IgM and fungal melanin.

## 7 Conclusion and future perspectives

Historically underrecognized relative to the high-affinity, class-switched IgG classes, the IgM antibody class is now gaining recognition as an indispensable component of host antifungal defense. Emerging evidence demonstrates the unique and non-redundant functions of both natural and acquired IgM isotypes in the immunological surveillance of fungal pathogens, primarily via their capacity to detect conserved polysaccharide motifs such as β-glucans and chitin on fungal cell walls. These interactions represent a critical axis within the interface of innate and adaptive immunity, with substantial implications for fungal disease diagnosis, prophylaxis, and treatment. Contemporary immunological research has delineated IgM as a polyvalent effector, whose pentameric structure facilitates high-avidity engagement with repetitive glycan determinants on fungal cell wall. This configuration not only potentiates complement activation but also orchestrates opsonization, thereby enhancing phagocytic clearance by immune phagocytic cells. Notably, natural IgM, produced in the absence of prior antigenic stimulation, functions as a frontline immunological battalion, being ever present and able to rapidly coordinate early pathogen containment while simultaneously shaping the trajectory of the adaptive immune response, including T helper cell polarization. The presence of long-lived IgM+ memory B cells further challenges traditional paradigms of immunological memory, suggesting a more refined model of persistent antifungal surveillance.

Future directions in IgM research point toward transformative applications in diagnostic and therapeutic domains. Although IgM-based serodiagnostics are established for certain endemic mycoses (e.g., *Coccidioides* spp.) (137), their broader utility remains underexploited. Enhancing the sensitivity and specificity of assays targeting anti-glycan IgM, particularly for invasive fungal infections, holds promise for improving early detection, especially among immunocompromised hosts. Nonetheless, distinguishing between transient IgM responses and persistent titers in chronic or latent infections presents an ongoing challenge.

From the aspect of therapeutic strategies, the robust effector mechanisms mediated by IgM offer fertile ground for passive immunotherapeutic strategies. Monoclonal IgM antibodies directed against structurally conserved fungal glycans may serve as adjunctive agents alongside antifungal pharmacotherapies, particularly in settings marked by increasing antifungal resistance. Biological based engineering IgM antibodies for enhanced *in vivo* stability, facilitate tissue penetration, and glycan specificity could substantially augment host immunity. Moreover, the molecular principles governing IgM-glycan recognition are catalyzing novel vaccine design paradigms. Vaccines that preferentially elicit durable IgM responses against pan- or universal- fungal glycan epitopes may confer broad-spectrum, preemptive immunity. The potential development of pan-protective antibodies targeting these conserved fungal cell wall components represents a significant opportunity for prophylactic and therapeutic interventions in immunocompromised individuals who are particularly vulnerable to opportunistic fungal infections (138, 139). We have also assembled supporting evidence regarding the role of IgM in preventing infections caused by clinically important pathogenic fungi, as mentioned in **Table 1**.

**Table 1:** The table summarizes these clinically important fungi -specific host IgM interactions.

Fungal pathogen	Key fungal antigens targeted by IgM	Primary IgM-mediated effector functions	Clinical significance/key findings
*Pneumocystis* spp. Selected reference (58):	β-1,3-glucan, Chitin	- Opsonization for dendritic cell uptake - Enhancement of dendritic cell migration - Required for Th2/Th17 differentiation - Guides B-cell class switching	Protective: Natural IgM is crucial for early innate defense and for shaping the subsequent protective adaptive T-cell response.
*Candida albicans* Selected references (140, 141):	β-glucan, Mannan, Complement Receptor 3-related protein (CR3-RP)	- Complement activation - Opsonophagocytosis - Inhibition of epithelial adherence & invasion	Protective: Elevated IgM indicates recent/ongoing infection. Antibodies enhance phagocytosis and block virulence mechanisms.
*Cryptococcus neoformans* Selected references (69, 142, 143):	Glucuronoxylomannan (GXM), β-glucan (Laminarin)	- Inhibition of Titan cell formation - Augmentation of Th1 polarization - Enhances macrophage recruitment & phagocytosis - Prevents dissemination to the central nervous system	Protective: IgM deficiency is linked to increased susceptibility and brain dissemination. IgM directly inhibits the key virulence transition to Titan cells by altering gene expression.
*Histoplasma capsulatum* Selected references: (89–91)	Histone 2B-like protein (H2B) and Heat Shock Protein	- Reduction of fungal burden - Decreased pulmonary inflammation	Protective: Specific IgM mAbs against H2B are protective in murine models, reducing fungal load and prolonging survival.

In summary, the repositioning of IgM as a central mediator in antifungal immunity marks a significant conceptual shift in host-pathogen immunology. Beyond the role of a bystander, IgM is emerging as a multifaceted sentinel and rapid responder to fungal invaders and these remarkable molecules have a profound relevance to diagnostics, immunotherapy, and vaccinology. Continued exploration into the structural, functional, and translational dimensions of IgM-mediated antifungal defense holds considerable potential for addressing the escalating global burden of fungal diseases.

## Supplementary material

Supplementary Figure 1 Sequence alignment analysis to generate structural model of MAbCC5. Multiple sequence alignments of mouse B cell receptor (PDB ID: 6EMA), AAA, light chain (GenBank: AAA63380.1), and heavy chain GenBank: (AMN90557.1) using Clustral Omega. The residues contributed to CDR1, CDR2, and CDR3 are highlighted in red, grey, and blue, respectively.

Supplementary Table 1 The amino acid sequences applied in the structural modeling process for MAb CC5.

## References

[1] Hughes AL. Evolution of the host defense system. In: Kaufmann SHE Sher A Ahmed R, editors. Immunology of infectious diseases. Washington, DC, United States: ASM Press Washington, DC (2001). p. 67–75.

[2] Keyt BA Baliga R Sinclair AM Carroll SF Peterson MS. Structure, function, and therapeutic use of IgM antibodies. Antibodies (Basel). (2020) 9:53. doi: 10.3390/antib9040053, PMID: 33066119 PMC7709107

[3] Jones K Savulescu AF Brombacher F Hadebe S. Immunoglobulin M in health and diseases: How far have we come and what next? Front Immunol. (2020) 11:595535. doi: 10.3389/fimmu.2020.595535, PMID: 33193450 PMC7662119

[4] Gong S Ruprecht RM. Immunoglobulin M: An ancient antiviral weapon - rediscovered. Front Immunol. (2020) 11:1943. doi: 10.3389/fimmu.2020.01943, PMID: 32849652 PMC7432194

[5] Michaud E Mastrandrea C Rochereau N Paul S. Human secretory IgM: An elusive player in mucosal immunity. Trends Immunol. (2020) 41:141–56. doi: 10.1016/j.it.2019.12.005, PMID: 31928913

[6] Rothstein TL Griffin DO Holodick NE Quach TD Kaku H. Human B-1 cells take the stage. Ann N Y Acad Sci. (2013) 1285:97–114. doi: 10.1111/nyas.12137, PMID: 23692567 PMC4429725

[7] Baumgarth N. B-1 cell heterogeneity and the regulation of natural and antigen-induced IgM production. Front Immunol. (2016) 7:324. doi: 10.3389/fimmu.2016.00324, PMID: 27667991 PMC5016532

[8] Köhler JR Casadevall A Perfect J. The spectrum of fungi that infects humans. Cold Spring Harb Perspect Med. (2014) 5:a019273. doi: 10.1101/cshperspect.a019273, PMID: 25367975 PMC4292074

[9] Rokas A. Evolution of the human pathogenic lifestyle in fungi. Nat Microbiol. (2022) 7:607–19. doi: 10.1038/s41564-022-01112-0, PMID: 35508719 PMC9097544

[10] Nosanchuk JD. mGem: A quarter century with the Pirofski-Casadevall damage response framework-a dynamic construct for understanding microbial pathogenesis. mBio. (2025) 16:e0294524. doi: 10.1128/mbio.02945-24, PMID: 39932289 PMC11898690

[11] Proctor DM Drummond RA Lionakis MS Segre JA. One population, multiple lifestyles: Commensalism and pathogenesis in the human mycobiome. Cell Host Microbe. (2023) 31:539–53. doi: 10.1016/j.chom.2023.02.010, PMID: 37054674 PMC10155287

[12] Fidel PL Jr. Yano J Esher SK Noverr MC. Applying the host-microbe damage response framework to *Candida* pathogenesis: Current and prospective strategies to reduce Damage. J Fungi (Basel). (2020) 6:35. doi: 10.3390/jof6010035, PMID: 32168864 PMC7151217

[13] Pruksaphon K Amsri A Jeenkeawpieam J Thammasit P Nosanchuk JD Youngchim S. The microbial damage and host response framework: lesson learned from pathogenic survival trajectories and immunoinflammatory responses of *Talaromyces marneffei* infection. Front Immunol. (2024) 15:1448729. doi: 10.3389/fimmu.2024.1448729, PMID: 39188728 PMC11345217

[14] Iliev ID Brown GD Bacher P Gaffen SL Heitman J Klein BS. Focus on fungi. Cell. (2024) 187:5121–7. doi: 10.1016/j.cell.2024.08.016, PMID: 39303681 PMC11722117

[15] Gow NA Netea MG Munro CA Ferwerda G Bates S Mora-Montes HM. Immune recognition of *Candida albicans* beta-glucan by dectin-1. J Infect Dis. (2007) 196:1565–71. doi: 10.1086/523110, PMID: 18008237 PMC2655640

[16] Villalobos-Duno HL Barreto LA Alvarez-Aular Á Mora-Montes HM Lozoya-Pérez NE Franco B. Comparison of cell wall polysaccharide composition and structure between strains of *Sporothrix schenckii* and *Sporothrix brasiliensis* . Front Microbiol. (2021) 12:726958. doi: 10.3389/fmicb.2021.726958, PMID: 34616384 PMC8489378

[17] Daskalov A. Emergence of the fungal immune system. iScience. (2023) 26:106793. doi: 10.1016/j.isci.2023.106793, PMID: 37213230 PMC10197012

[18] Dickman MB Figueiredo P. Comparative pathobiology of fungal pathogens of plants and animals. PloS Pathog. (2011) 7:e1002324. doi: 10.1371/journal.ppat.1002324, PMID: 22194681 PMC3240592

[19] Park YD Williamson PR. Masking the pathogen: Evolutionary strategies of fungi and their bacterial counterparts. J Fungi (Basel). (2015) 1:397–421. doi: 10.3390/jof1030397, PMID: 29376918 PMC5753132

[20] Snarr BD Qureshi ST Sheppard DC. Immune recognition of fungal polysaccharides. J Fungi (Basel). (2017) 3:47. doi: 10.3390/jof3030047, PMID: 29371564 PMC5715945

[21] Garcia-Rubio R de Oliveira HC Rivera J Trevijano-Contador N. The fungal cell wall: *Candida*, *Cryptococcus*, and *Aspergillus* species. Front Microbiol. (2019) 10:2993. doi: 10.3389/fmicb.2019.02993, PMID: 31993032 PMC6962315

[22] Shende R Wong SSW Meitei HT Lal G Madan T Aimanianda V. Protective role of host complement system in *Aspergillus fumigatus* infection. Front Immunol. (2022) 13:978152. doi: 10.3389/fimmu.2022.978152, PMID: 36211424 PMC9539816

[23] WHO FPPL World Health Organization. WHO fungal priority pathogens list to guide research, development and public health action (ISBN 978-92-4-006024-1). World Health Organization (2022). Available online at: https://www.who.int/publications/i/item/9789240060241 (Accessed October 24, 2025).

[24] Brown GD Ballou ER Bates S Bignell EM Borman AM Brand AC. The pathobiology of human fungal infections. Nat Rev Microbiol. (2024) 22:687–704. doi: 10.1038/s41579-024-01062-w, PMID: 38918447

[25] Sørensen V Sundvold V Michaelsen TE Sandlie I. Polymerization of IgA and IgM: roles of Cys309/Cys414 and the secretory tailpiece. J Immunol. (1999) 162:3448–55. doi: 10.4049/jimmunol.162.6.3448, PMID: 10092800

[26] Giannone C Mess X He R Chelazzi MR Mayer A Bakunts A. How J-chain ensures the assembly of immunoglobulin IgM pentamers. EMBO J. (2025) 44:505–33. doi: 10.1038/s44318-024-00317-9, PMID: 39632981 PMC11729874

[27] Wiersma EJ Chen F Bazin R Collins C Painter RH Lemieux R. Analysis of IgM structures involved in J chain incorporation. J Immunol. (1997) 158:1719–26. doi: 10.4049/jimmunol.158.4.1719, PMID: 9029108

[28] Li Y Wang G Li N Wang Y Zhu Q Chu H. Structural insights into immunoglobulin M. Science. (2020) 367:1014–7. doi: 10.1126/science.aaz5425, PMID: 32029689

[29] Watson MJ Mundorff CC Lynch EM Kollman JM Kearney JF Guttman M. Defining the features of complement-active IgM. J Mol Biol. (2025) 437:169104. doi: 10.1016/j.jmb.2025.169104, PMID: 40154915 PMC12040574

[30] Chen Q Menon R Calder LJ Tolar P Rosenthal PB. Cryomicroscopy reveals the structural basis for a flexible hinge motion in the immunoglobulin M pentamer. Nat Commun. (2022) 13:6314. doi: 10.1038/s41467-022-34090-2, PMID: 36274064 PMC9588798

[31] Su Q Chen M Shi Y Zhang X Huang G Huang B. Cryo-EM structure of the human IgM B cell receptor. Science. (2022) 377:875–80. doi: 10.1126/science.abo3923, PMID: 35981043

[32] Oskam N Ooijevaar-de Heer P Derksen NIL Kruithof S de Taeye SW Vidarsson G. At critically low antigen densities, IgM hexamers outcompete both IgM pentamers and IgG1 for human complement deposition and complement-dependent cytotoxicity. J Immunol. (2022) 209:16–25. doi: 10.4049/jimmunol.2101196, PMID: 35705253

[33] Grönwall C Vas J Silverman GJ. Protective roles of natural IgM antibodies. Front Immunol. (2012) 3:66. doi: 10.3389/fimmu.2012.00066, PMID: 22566947 PMC3341951

[34] Buchner J Sitia R Svilenov HL. Understanding IgM structure and biology to engineer new antibody therapeutics. BioDrugs. (2025) 39:347–57. doi: 10.1007/s40259-025-00720-6, PMID: 40237925 PMC12031937

[35] Allman D Pillai S. Peripheral B cell subsets. Curr Opin Immunol. (2008) 20:149–57. doi: 10.1016/j.coi.2008.03.014, PMID: 18434123 PMC2532490

[36] Pone EJ Hernandez-Davies JE Jan S Silzel E Felgner PL Davies DH. Multimericity amplifies the synergy of BCR and TLR4 for B cell activation and antibody class switching. Front Immunol. (2022) 13:882502. doi: 10.3389/fimmu.2022.882502, PMID: 35663959 PMC9161726

[37] Mukherjee S Karmakar S Babu SP. TLR2 and TLR4 mediated host immune responses in major infectious diseases: a review. Braz J Infect Dis. (2016) 20:193–204. doi: 10.1016/j.bjid.2015.10.011, PMID: 26775799 PMC9427569

[38] Komegae EN Grund LZ Lopes-Ferreira M Lima C. TLR2, TLR4 and the MyD88 signaling are crucial for the *in vivo* generation and the longevity of long-lived antibody-secreting cells. PloS One. (2013) 8:e71185. doi: 10.1371/journal.pone.0071185, PMID: 23940714 PMC3733974

[39] Cervantes-Barragán L Gil-Cruz C Pastelin-Palacios R Lang KS Isibasi A Ludewig B. TLR2 and TLR4 signaling shapes specific antibody responses to *Salmonella typhi* antigens. Eur J Immunol. (2009) 39:126–35. doi: 10.1002/eji.200838185, PMID: 19130558

[40] Ferreira Silva M Salomão Lopes C Batista Ferreira França F Lucas Pires Ramos E Maria Santiago F Roberto Mineo J. Role of TLR2/MyD88 in the production of specific IgM and IgG antibodies during the immunization of mice against. Neospora caninum Vaccine. (2022) 40:5860–7. doi: 10.1016/j.vaccine.2022.08.067, PMID: 36075796

[41] Liu X Zhao Y Qi H. T-independent antigen induces humoral memory through germinal centers. J Exp Med. (2022) 219:e20210527. doi: 10.1084/jem.20210527, PMID: 35019947 PMC8759593

[42] Nera KP Kohonen P Narvi E Peippo A Mustonen L Terho P. Loss of Pax5 promotes plasma cell differentiation. Immunity. (2006) 24:283–93. doi: 10.1016/j.immuni.2006.02.003, PMID: 16546097

[43] Brynjolfsson SF Persson Berg L Olsen Ekerhult T Rimkute I Wick MJ Mårtensson IL. Long-lived plasma cells in mice and men. Front Immunol. (2018) 9: 2673. doi: 10.3389/fimmu.2018.02673, PMID: 30505309 PMC6250827

[44] Liu J Wang Y Xiong E Hong R Lu Q Ohno H. Role of the IgM Fc receptor in immunity and tolerance. Front Immunol. (2019) 10:529. doi: 10.3389/fimmu.2019.00529, PMID: 30967868 PMC6438924

[45] Baumgarth N Tung JW Herzenberg LA. Inherent specificities in natural antibodies: a key to immune defense against pathogen invasion. Springer Semin Immunopathol. (2005) 26:347–62. doi: 10.1007/s00281-004-0182-2, PMID: 15633017

[46] Vale AM Nobrega A Schroeder HW Jr. The role of evolutionarily conserved germ-line DH sequence in B-1 cell development and natural antibody production. Ann N Y Acad Sci. (2015) 1362:48–56. doi: 10.1111/nyas.12808, PMID: 26104486 PMC4679679

[47] Kubagawa H Honjo K Ohkura N Sakaguchi S Radbruch A Melchers F. Functional roles of the IgM Fc receptor in the immune system. Front Immunol. (2019) 10:945. doi: 10.3389/fimmu.2019.00945, PMID: 31130948 PMC6509151

[48] Rubtsov AV Swanson CL Troy S Strauch P Pelanda R Torres RM. TLR agonists promote marginal zone B cell activation and facilitate T-dependent IgM responses. J Immunol. (2008) 180:3882–8. doi: 10.4049/jimmunol.180.6.3882, PMID: 18322196

[49] Sharma J Mudalagiriyappa S Nanjappa SG. T cell responses to control fungal infection in an immunological memory lens. Front Immunol. (2022) 13:905867. doi: 10.3389/fimmu.2022.905867, PMID: 36177012 PMC9513067

[50] Capolunghi F Rosado MM Sinibaldi M Aranburu A Carsetti R. Why do we need IgM memory B cells? Immunol Lett. (2013) 152:114–20. doi: 10.1016/j.imlet.2013.04.007, PMID: 23660557

[51] Bohannon C Powers R Satyabhama L Cui A Tipton C Michaeli M. Long-lived antigen-induced IgM plasma cells demonstrate somatic mutations and contribute to long-term protection. Nat Commun. (2016) 7:11826. doi: 10.1038/ncomms11826, PMID: 27270306 PMC4899631

[52] Horiuchi K Imai K Mitsui-Sekinaka K Yeh TW Ochs HD Durandy A. Analysis of somatic hypermutations in the IgM switch region in human B cells. J Allergy Clin Immunol. (2014) 134:411–9. doi: 10.1016/j.jaci.2014.02.043, PMID: 24836470

[53] Gow NAR Casadevall A Fang W. Top five unanswered questions in fungal cell surface research. Cell Surf. (2023) 10:100114. doi: 10.1016/j.tcsw.2023.100114, PMID: 38024560 PMC10654581

[54] Lionakis MS Drummond RA Hohl TM. Immune responses to human fungal pathogens and therapeutic prospects. Nat Rev Immunol. (2023) 23:433–52. doi: 10.1038/s41577-022-00826-w, PMID: 36600071 PMC9812358

[55] Chang Q Abadi J Rosenberg M Pirofski LA. VH3 gene expression in children with HIV infection. J Infect. (2004) 49:274–82. doi: 10.1016/j.jinf.2004.04.007, PMID: 15474624

[56] Scamurra RW Miller DJ Dahl L Abrahamsen M Kapur V Wahl SM. Impact of HIV-1 infection on VH3 gene repertoire of naive human B cells. J Immunol. (2000) 164:5482–91. doi: 10.4049/jimmunol.164.10.5482, PMID: 10799916

[57] Zhou ZH Zhang Y Hu YF Wahl LM Cisar JO Notkins AL. The broad antibacterial activity of the natural antibody repertoire is due to polyreactive antibodies. Cell Host Microbe. (2007) 1:51–61. doi: 10.1016/j.chom.2007.01.002, PMID: 18005681 PMC2212603

[58] Rapaka RR Ricks DM Alcorn JF Chen K Khader SA Zheng M. Conserved natural IgM antibodies mediate innate and adaptive immunity against the opportunistic fungus. Pneumocystis murina J Exp Med. (2010) 207:2907–19. doi: 10.1084/jem.20100034, PMID: 21149550 PMC3005228

[59] Doron I Kusakabe T Iliev ID. Immunoglobulins at the interface of the gut mycobiota and anti-fungal immunity. Semin Immunol. (2023) 67:101757. doi: 10.1016/j.smim.2023.101757, PMID: 37003056 PMC10192079

[60] Oliveira M Oliveira D Lisboa C Boechat JL Delgado L. Clinical manifestations of human exposure to fungi. J Fungi (Basel). (2023) 9:381. doi: 10.3390/jof9030381, PMID: 36983549 PMC10052331

[61] Trevijano-Contador N de Oliveira HC Malacatus-Bravo C Sarai V Cuesta I Rodrigues ML. Effects of human immunoglobulin A on *Cryptococcus neoformans* morphology and gene expression. Microbiol Spectr. (2025) 13:e0200824. doi: 10.1128/spectrum.02008-24, PMID: 39982066 PMC11960444

[62] Pirofski LA. Virulence profile: liise-anne pirofski. Virulence. (2018) 9:275–7. doi: 10.1080/21505594.2017.1382277, PMID: 28956699 PMC5990341

[63] Kelsoe G Cerny J. Reciprocal expansions of idiotypic and anti-idiotypic clones following antigen stimulation. Nature. (1979) 279:333–4. doi: 10.1038/279333a0, PMID: 313014

[64] Civello A. On the genesis of the idiotypic network theory. J Hist Biol. (2013) 46:125–58. doi: 10.1007/s10739-012-9346-4, PMID: 23207664

[65] Subramaniam KS Datta K Marks MS Pirofski LA. Improved survival of mice deficient in secretory immunoglobulin M following systemic infection with *Cryptococcus neoformans* . Infect Immun. (2010) 78:441–52. doi: 10.1128/iai.00506-09, PMID: 19901068 PMC2798234

[66] Pirofski LA. Of mice and men, revisited: new insights into an ancient molecule from studies of complement activation by *Cryptococcus neoformans* . Infect Immun. (2006) 74:3079–84. doi: 10.1128/iai.00431-06, PMID: 16714535 PMC1479240

[67] Biswas PS. Vaccine-induced immunological memory in invasive fungal infections - A dream so close yet so far. Front Immunol. (2021) 12:671068. doi: 10.3389/fimmu.2021.671068, PMID: 33968079 PMC8096976

[68] Fleuridor R Zhong Z Pirofski L. A human IgM monoclonal antibody prolongs survival of mice with lethal cryptococcosis. J Infect Dis. (1998) 178:1213–6. doi: 10.1086/515688, PMID: 9806064

[69] Trevijano-Contador N Pianalto KM Nichols CB Zaragoza O Alspaugh JA Pirofski LA. Human IgM inhibits the formation of Titan-like cells in *Cryptococcus neoformans* . Infect Immun. (2020) 88:e00046–20. doi: 10.1128/iai.00046-20, PMID: 31988178 PMC7093138

[70] Yoon HA Nakouzi A Chang CC Kuniholm MH Carreño LJ Wang T. Association between plasma antibody responses and risk for *Cryptococcus*-associated immune reconstitution inflammatory syndrome. J Infect Dis. (2019) 219:420–8. doi: 10.1093/infdis/jiy447, PMID: 30010905 PMC6325352

[71] Subramaniam K Metzger B Hanau LH Guh A Rucker L Badri S. IgM(+) memory B cell expression predicts HIV-associated cryptococcosis status. J Infect Dis. (2009) 200:244–51. doi: 10.1086/599318, PMID: 19527168 PMC2805277

[72] Skipper C Abassi M Boulware DR. Diagnosis and management of central nervous system cryptococcal infections in HIV-Infected adults. J Fungi (Basel). (2019) 5:65. doi: 10.3390/jof5030065, PMID: 31330959 PMC6787675

[73] Pruksaphon K Amsri A Thammasit P Nosanchuk JD Aiumurai P Youngchim S. Diagnostic performances of an in-house immunochromatography test based on the monoclonal antibody 18B7 to gucuronoxylomannan for clinical suspected cryptococcosis: a large-scale prototype evaluation in Northern Thailand. Mycopathologia. (2024) 189:75. doi: 10.1007/s11046-024-00882-x, PMID: 39120647 PMC11517805

[74] Hlupeni A Nakouzi A Wang T Boyd KF Makadzange TA Ndhlovu CE. Antibody responses in HIV-infected patients with advanced immunosuppression and asymptomatic cryptococcal antigenemia. Open Forum Infect Dis. (2019) 6:ofy333. doi: 10.1093/ofid/ofy333, PMID: 30648127 PMC6329905

[75] Fu M Ren J An J Liu Y Li W. *In situ* IgM production and clonal expansion of B-1 cells in peritoneal cavity promote elimination of C. albicans infection in IgH transgenic mice with VH derived from a natural antibody. PloS One. (2013) 8:e60779. doi: 10.1371/journal.pone.0060779, PMID: 23565274 PMC3614557

[76] Li W Fu M An JG Xing Y Zhang P Zhang X. Host defence against C. albicans infections in IgH transgenic mice with V(H) derived from a natural anti-keratin antibody. Cell Microbiol. (2007) 9:306–15. doi: 10.1111/j.1462-5822.2006.00786.x, PMID: 16925788

[77] Han Y Cutler JE. Antibody response that protects against disseminated candidiasis. Infect Immun. (1995) 63:2714–9. doi: 10.1128/iai.63.7.2714-2719.1995, PMID: 7790089 PMC173363

[78] Han Y Cutler JE. Assessment of a mouse model of neutropenia and the effect of an anti-candidiasis monoclonal antibody in these animals. J Infect Dis. (1997) 175:1169–75. doi: 10.1086/516455, PMID: 9129081

[79] Han Y Riesselman MH Cutler JE. Protection against candidiasis by an immunoglobulin G3 (IgG3) monoclonal antibody specific for the same mannotriose as an IgM protective antibody. Infect Immun. (2000) 68:1649–54. doi: 10.1128/iai.68.3.1649-1654.2000, PMID: 10678984 PMC97325

[80] Han Y. Efficacy of combination immunotherapy of IgM MAb B6.1 and amphotericin B against disseminated candidiasis. Int Immunopharmacol. (2010) 10:1526–31. doi: 10.1016/j.intimp.2010.08.027, PMID: 20840836

[81] Johnson MA Cartmell J Weisser NE Woods RJ Bundle DR. Molecular recognition of *Candida albicans* (1->2)-β-mannan oligosaccharides by a protective monoclonal antibody reveals the immunodominance of internal saccharide residues. J Biol Chem. (2012) 287:18078–90. doi: 10.1074/jbc.M112.355578, PMID: 22493450 PMC3365708

[82] Han Y Kozel TR Zhang MX MacGill RS Carroll MC Cutler JE. Complement is essential for protection by an IgM and an IgG3 monoclonal antibody against experimental, hematogenously disseminated candidiasis. J Immunol. (2001) 167:1550–7. doi: 10.4049/jimmunol.167.3.1550, PMID: 11466376

[83] Caesar-TonThat TC Cutler JE. A monoclonal antibody to *Candida albicans* enhances mouse neutrophil candidacidal activity. Infect Immun. (1997) 65:5354–7. doi: 10.1128/iai.65.12.5354-5357.1997, PMID: 9393840 PMC175773

[84] Konsoula A Tsioutis C Markaki I Papadakis M Agouridis AP Spernovasilis N. Lomentospora prolificans: An emerging opportunistic fungal pathogen. Microorganisms. (2022) 10:1317. doi: 10.3390/microorganisms10071317, PMID: 35889036 PMC9316904

[85] Brena S Cabezas-Olcoz J Moragues MD Fernández de Larrinoa I Domínguez A Quindós G. Fungicidal monoclonal antibody C7 interferes with iron acquisition in *Candida albicans* . Antimicrob Agents Chemother. (2011) 55:3156–63. doi: 10.1128/aac.00892-10, PMID: 21518848 PMC3122412

[86] Li N Zhang J Yu F Ye F Tan W Hao L. Garlic-derived quorum sensing inhibitors: A novel strategy against fungal resistance. Drug Des Devel Ther. (2024) 18:6413–26. doi: 10.2147/dddt.S503302, PMID: 39749188 PMC11693938

[87] Chaturvedi AK Kumar R Kumar A Shukla PK. A monoclonal IgM directed against immunodominant catalase B of cell wall of *Aspergillus fumigatus* exerts anti-A. fumigatus activities. Mycoses. (2009) 52:524–33. doi: 10.1111/j.1439-0507.2008.01635.x, PMID: 18983426

[88] Appel E Vallon-Eberhard A Rabinkov A Brenner O Shin I Sasson K. Therapy of murine pulmonary aspergillosis with antibody-alliinase conjugates and alliin. Antimicrob Agents Chemother. (2010) 54:898–906. doi: 10.1128/aac.01267-09, PMID: 19949059 PMC2812126

[89] Shi L Albuquerque PC Lazar-Molnar E Wang X Santambrogio L Gácser A. A monoclonal antibody to *Histoplasma capsulatum* alters the intracellular fate of the fungus in murine macrophages. Eukaryot Cell. (2008) 7:1109–17. doi: 10.1128/ec.00036-08, PMID: 18487350 PMC2446677

[90] Nosanchuk JD Steenbergen JN Shi L Deepe GS Jr. Casadevall A. Antibodies to a cell surface histone-like protein protect against *Histoplasma capsulatum* . J Clin Invest. (2003) 112:1164–75. doi: 10.1172/jci19361, PMID: 14561701 PMC213494

[91] Guimarães AJ Frases S Gomez FJ Zancopé-Oliveira RM Nosanchuk JD. Monoclonal antibodies to heat shock protein 60 alter the pathogenesis of *Histoplasma capsulatum* . Infect Immun. (2009) 77:1357–67. doi: 10.1128/iai.01443-08, PMID: 19179416 PMC2663142

[92] Tsoni SV Kerrigan AM Marakalala MJ Srinivasan N Duffield M Taylor PR. Complement C3 plays an essential role in the control of opportunistic fungal infections. Infect Immun. (2009) 77:3679–85. doi: 10.1128/IAI.00233-09, PMID: 19581397 PMC2738051

[93] Cohen DG Wingert RA. You shall not pass: how complement C5 mediated antifungal immunity blocks systemic candidiasis and preserves renal tissue barriers. Tissue Barriers. (2024) 12:2257110. doi: 10.1080/21688370.2023.2257110, PMID: 37794527 PMC11262218

[94] Harpf V Rambach G Würzner R Lass-Flörl C Speth C. *Candida* and complement: New aspects in an old battle. Front Immunol. (2020) 11:1471. doi: 10.3389/fimmu.2020.01471, PMID: 32765510 PMC7381207

[95] Zaragoza O Casadevall A. Monoclonal antibodies can affect complement deposition on the capsule of the pathogenic fungus *Cryptococcus neoformans* by both classical pathway activation and steric hindrance. Cell Microbiol. (2006) 8:1862–76. doi: 10.1111/j.1462-5822.2006.00753.x, PMID: 16824038

[96] Levitz SM. Innate recognition of fungal cell walls. PloS Pathog. (2010) 6:e1000758. doi: 10.1371/journal.ppat.1000758, PMID: 20421940 PMC2858700

[97] Genster N Præstekjær Cramer E Rosbjerg A Pilely K Cowland JB Garred P. Ficolins Promote Fungal Clearance *in vivo* and Modulate the Inflammatory Cytokine Response in Host Defense against *Aspergillus fumigatus* . J Innate Immun. (2016) 8:579–88. doi: 10.1159/000447714, PMID: 27467404 PMC6738752

[98] Bidula S Sexton DW Yates M Abdolrasouli A Shah A Wallis R. H-ficolin binds *Aspergillus fumigatus* leading to activation of the lectin complement pathway and modulation of lung epithelial immune responses. Immunology. (2015) 146:281–91. doi: 10.1111/imm.12501, PMID: 26133042 PMC4582969

[99] Bidula S Kenawy H Ali YM Sexton D Schwaeble WJ Schelenz S. Role of ficolin-A and lectin complement pathway in the innate defense against pathogenic Aspergillus species. Infect Immun. (2013) 81:1730–40. doi: 10.1128/IAI.00032-13, PMID: 23478320 PMC3647983

[100] Ma L Li D Wen Y Shi D. Advances in understanding the role of pentraxin-3 in lung infections. Front Immunol. (2025) 16:1575968. doi: 10.3389/fimmu.2025.1575968, PMID: 40313930 PMC12043646

[101] Patel P Kearney JF. Immunological outcomes of antibody binding to glycans shared between microorganisms and mammals. J Immunol. (2016) 197:4201–9. doi: 10.4049/jimmunol.1600872, PMID: 27864551 PMC5119654

[102] Amsri A Pruksaphon K Thammasit P Nosanchuk JD Youngchim S. Adaptation to an amoeba host drives selection of virulence-associated traits and genetic variation in saprotrophic *Candida albicans* . Front Cell Infect Microbiol. (2024) 14:1367656. doi: 10.3389/fcimb.2024.1367656, PMID: 38550616 PMC10976851

[103] Pruksaphon K Nosanchuk JD Thammasit P Pongpom M Youngchim S. Interaction of *Talaromyces marneffei* with free living soil amoeba as a model of fungal pathogenesis. Front Cell Infect Microbiol. (2022) 12:1023067. doi: 10.3389/fcimb.2022.1023067, PMID: 36262181 PMC9574045

[104] Hernández O Tamayo D Torres I McEwen JG García AM. Kinetic analysis of gene expression during mycelium to yeast transition and yeast to mycelium germination in *Paracoccidioides brasiliensis* . Biomedica. (2011) 31:570–9. doi: 10.1590/s0120-41572011000400012, PMID: 22674368

[105] Zaragoza O Chrisman CJ Castelli MV Frases S Cuenca-Estrella M Rodríguez-Tudela JL. Capsule enlargement in *Cryptococcus neoformans* confers resistance to oxidative stress suggesting a mechanism for intracellular survival. Cell Microbiol. (2008) 10:2043–57. doi: 10.1111/j.1462-5822.2008.01186.x, PMID: 18554313 PMC4405381

[106] Kulig K Rapala-Kozik M Karkowska-Kuleta J. Extracellular vesicle production: A bidirectional effect in the interplay between host and *Candida* fungi. Curr Res Microb Sci. (2024) 7:100255. doi: 10.1016/j.crmicr.2024.100255, PMID: 39040088 PMC11260599

[107] Xiao W Lu H Jiang B Zheng Y Chen P Liu X. Virulence factors released by extracellular vesicles from *Cryptococcus neoformans* . Front Cell Infect Microbiol. (2025) 15:1572520. doi: 10.3389/fcimb.2025.1572520, PMID: 40438241 PMC12116589

[108] Pruksaphon K Amsri A Thammasit P Nosanchuk JD Youngchim S. Extracellular vesicles derived from *Talaromyces marneffei* contain immunogenic compounds and modulate THP-1 macrophage responses. Front Immunol. (2023) 14:1192326. doi: 10.3389/fimmu.2023.1192326, PMID: 37457708 PMC10339390

[109] Baltazar LM Ribeiro GF Freitas GJ Queiroz-Junior CM Fagundes CT Chaves-Olórtegui C. Protective response in experimental paracoccidioidomycosis elicited by extracellular vesicles containing antigens of *Paracoccidioides brasiliensis* . Cells. (2021) 10:1813. doi: 10.3390/cells10071813, PMID: 34359982 PMC8304155

[110] Del Bino L Romano MR. Role of carbohydrate antigens in antifungal glycoconjugate vaccines and immunotherapy. Drug Discov Today Technol. (2020) 38:45–55. doi: 10.1016/j.ddtec.2021.02.002, PMID: 34895640

[111] Hudson KL Bartlett GJ Diehl RC Agirre J Gallagher T Kiessling LL. Carbohydrate-aromatic interactions in proteins. J Am Chem Soc. (2015) 137:15152–60. doi: 10.1021/jacs.5b08424, PMID: 26561965 PMC4676033

[112] Spiwok V. CH/π Interactions in carbohydrate recognition. Molecules. (2017) 22:1038. doi: 10.3390/molecules22071038, PMID: 28644385 PMC6152320

[113] Mattox DE Bailey-Kellogg C. Comprehensive analysis of lectin-glycan interactions reveals determinants of lectin specificity. PloS Comput Biol. (2021) 17:e1009470. doi: 10.1371/journal.pcbi.1009470, PMID: 34613971 PMC8523061

[114] Stanković IM Blagojević Filipović JP Zarić SD. Carbohydrate - protein aromatic ring interactions beyond CH/π interactions: A protein data bank survey and quantum chemical calculations. Int J Biol Macromol. (2020) 157:1–9. doi: 10.1016/j.ijbiomac.2020.03.251, PMID: 32268187

[115] Nelson DL Cox MM. Carbohydrates and glycobiology. In: Lehninger principles of biochemistry, 7th ed. New York, United States: W.H. Freeman (2017). p. 375–423.

[116] Muthana SM Xia L Campbell CT Zhang Y Gildersleeve JC. Competition between serum IgG, IgM, and IgA anti-glycan antibodies. PloS One. (2015) 10:e0119298. doi: 10.1371/journal.pone.0119298, PMID: 25807519 PMC4373866

[117] McConnell SA Casadevall A. New insights into antibody structure with implications for specificity, variable region restriction and isotype choice. Nat Rev Immunol. (2025) 25:621–632. doi: 10.1038/s41577-025-01150-9, PMID: 40113994

[118] Janda A Bowen A Greenspan NS Casadevall A. Ig constant region effects on variable region structure and function. Front Microbiol. (2016) 7:22. doi: 10.3389/fmicb.2016.00022, PMID: 26870003 PMC4740385

[119] Sheriff S Chang CY Ezekowitz RA. Human mannose-binding protein carbohydrate recognition domain trimerizes through a triple alpha-helical coiled-coil. Nat Struct Biol. (1994) 1:789–94. doi: 10.1038/nsb1194-789, PMID: 7634089

[120] Woods RJ. Predicting the structures of glycans, glycoproteins, and their complexes. Chem Rev. (2018) 118:8005–24. doi: 10.1021/acs.chemrev.8b00032, PMID: 30091597 PMC6659753

[121] Vollmers HP Brändlein S. Natural IgM antibodies: the orphaned molecules in immune surveillance. Adv Drug Delivery Rev. (2006) 58:755–65. doi: 10.1016/j.addr.2005.08.007, PMID: 16820243

[122] Buettner MJ Shah SR Saeui CT Ariss R Yarema KJ. Improving immunotherapy through glycodesign. Front Immunol. (2018) 9:2485. doi: 10.3389/fimmu.2018.02485, PMID: 30450094 PMC6224361

[123] Kaur H Salunke DM. Antibody promiscuity: Understanding the paradigm shift in antigen recognition. IUBMB Life. (2015) 67:498–505. doi: 10.1002/iub.1397, PMID: 26177714

[124] Scherbinina SI Toukach PV. Three-dimensional structures of carbohydrates and where to find them. Int J Mol Sci. (2020) 21:7702. doi: 10.3390/ijms21207702, PMID: 33081008 PMC7593929

[125] Krishnan L Sahni G Kaur KJ Salunke DM. Role of antibody paratope conformational flexibility in the manifestation of molecular mimicry. Biophys J. (2008) 94:1367–76. doi: 10.1529/biophysj.107.108654, PMID: 18032557 PMC2212675

[126] Haji-Ghassemi O Blackler RJ Martin Young N Evans SV. Antibody recognition of carbohydrate epitopes†. Glycobiol. (2015) 25:920–52. doi: 10.1093/glycob/cwv037, PMID: 26033938

[127] Cummings RD Etzler M Hahn MG Darvill A Godula K Woods RJ. Glycan-recognizing probes as tools. In: Varki A Cummings RD Esko JD, editors. Essentials of glycobiology, 4th ed. New York, United States: Cold Spring Harbor Laboratory Press (2022). Available online at: https://www.ncbi.nlm.nih.gov/books/NBK579992/.

[128] Figueiredo ABC Fonseca FL Kuczera D Conte FP Arissawa M Rodrigues ML. Monoclonal Antibodies against cell wall chitooligomers as accessory tools for the control of cryptococcosis. Antimicrob Agents Chemother. (2021) 65:e0118121. doi: 10.1128/aac.01181-21, PMID: 34570650 PMC8597760

[129] Sievers F Wilm A Dineen D Gibson TJ Karplus K Li W. Fast, scalable generation of high-quality protein multiple sequence alignments using Clustal Omega. Mol Syst Biol. (2011) 7:539. doi: 10.1038/msb.2011.75, PMID: 21988835 PMC3261699

[130] Abramson J Adler J Dunger J Evans R Green T Pritzel A. Accurate structure prediction of biomolecular interactions with AlphaFold 3. Nature. (2024) 630:493–500. doi: 10.1038/s41586-024-07487-w, PMID: 38718835 PMC11168924

[131] Villeneuve S Souchon H Riottot MM Mazie JC Lei P Glaudemans CP. Crystal structure of an anti-carbohydrate antibody directed against *Vibrio cholerae* O1 in complex with antigen: molecular basis for serotype specificity. Proc Natl Acad Sci U S A. (2000) 97:8433–8. doi: 10.1073/pnas.060022997, PMID: 10880560 PMC26965

[132] Liu Y Yang X Gan J Chen S Xiao ZX Cao Y. CB-Dock2: improved protein-ligand blind docking by integrating cavity detection, docking and homologous template fitting. Nucleic Acids Res. (2022) 50:W159–w64. doi: 10.1093/nar/gkac394, PMID: 35609983 PMC9252749

[133] Mishra SK Koča J. Assessing the performance of MM/PBSA, MM/GBSA, and QM-MM/GBSA approaches on protein/carbohydrate complexes: Effect of implicit solvent models, QM methods, and entropic contributions. J Phys Chem B. (2018) 122:8113–21. doi: 10.1021/acs.jpcb.8b03655, PMID: 30084252

[134] Rosas AL Nosanchuk JD Casadevall A. Passive immunization with melanin-binding monoclonal antibodies prolongs survival of mice with lethal *Cryptococcus neoformans* infection. Infect Immun. (2001) 69:3410–2. doi: 10.1128/iai.69.5.3410-3412.2001, PMID: 11292764 PMC98300

[135] Liu S Youngchim S Zamith-Miranda D Nosanchuk JD. Fungal melanin and the mammalian immune system. J Fungi (Basel). (2021) 7:264. doi: 10.3390/jof7040264, PMID: 33807336 PMC8066723

[136] Nosanchuk JD Stark RE Casadevall A. Fungal melanin: What do we know about structure? Front Microbiol. (2015) 6:1463. doi: 10.3389/fmicb.2015.01463, PMID: 26733993 PMC4687393

[137] Donovan FM Zangeneh TT Malo J Galgiani JN. Top Questions in the diagnosis and treatment of coccidioidomycosis. Open Forum Infect Dis. (2017) 4:ofx197. doi: 10.1093/ofid/ofx197, PMID: 29670928 PMC5903411

[138] Rabaan AA Alfaraj AH Alshengeti A Alawfi A Alwarthan S Alhajri M. Antibodies to combat fungal infections: Development strategies and progress. Microorganisms. (2023) 11:671. doi: 10.3390/microorganisms11030671, PMID: 36985244 PMC10051215

[139] Levitz SM Huang H Ostroff GR Specht CA. Exploiting fungal cell wall components in vaccines. Semin Immunopathol. (2015) 37:199–207. doi: 10.1007/s00281-014-0460-6, PMID: 25404118 PMC4329074

[140] Bromuro C Posteraro B Murri R Fantoni M Tumbarello M Sanguinetti M. Identification of two anti-Candida antibodies associated with the survival of patients with candidemia. mBio. (2024) 15:e0276923. doi: 10.1128/mbio.02769-23, PMID: 38088540 PMC10790786

[141] Wich M Greim S Ferreira-Gomes M Krüger T Kniemeyer O Brakhage AA. Functionality of the human antibody response to. Candida albicans Virulence. (2021) 12:3137–48. doi: 10.1080/21505594.2021.2015116, PMID: 34923920 PMC8923069

[142] Subramaniam KS Datta K Quintero E Manix C Marks MS Pirofski LA. The absence of serum IgM enhances the susceptibility of mice to pulmonary challenge with Cryptococcus neoformans. J Immunol. (2010) 184:5755–67. doi: 10.4049/jimmunol.0901638, PMID: 20404271 PMC2885446

[143] Dufaud C Rivera J Rohatgi S Pirofski LA. Naïve B cells reduce fungal dissemination in Cryptococcus neoformans infected Rag1^-/-^ mice. Virulence. (2018) 9:173–84. doi: 10.1080/21505594.2017.1370529, PMID: 28837391 PMC5955176

