# Supplementary material for: Exploring the structural basis and functional immunodynamics of immunoglobulin M in host defense against fungal pathogens
Source: Front Immunol. 2025 Oct 30;16:1666690. doi: 10.3389/fimmu.2025.1666690 (PMC12611827; doi:10.3389/fimmu.2025.1666690)
Supplement: Supplementary Figure 1 — Sequence alignment analysis to generate structural model of MAbCC5. Multiple sequence alignments of mouse B cell receptor (PDB ID: 6EMA), AAA, light chain (GenBank: AAA63380.1), and heavy chain GenBank: (AMN90557.1) using Clustral Omega. The residues contributed to CDR1, CDR2, and CDR3 are highlighted in red, grey, and blue, respectively. [file DataSheet1.docx]

**Supplementary Information**


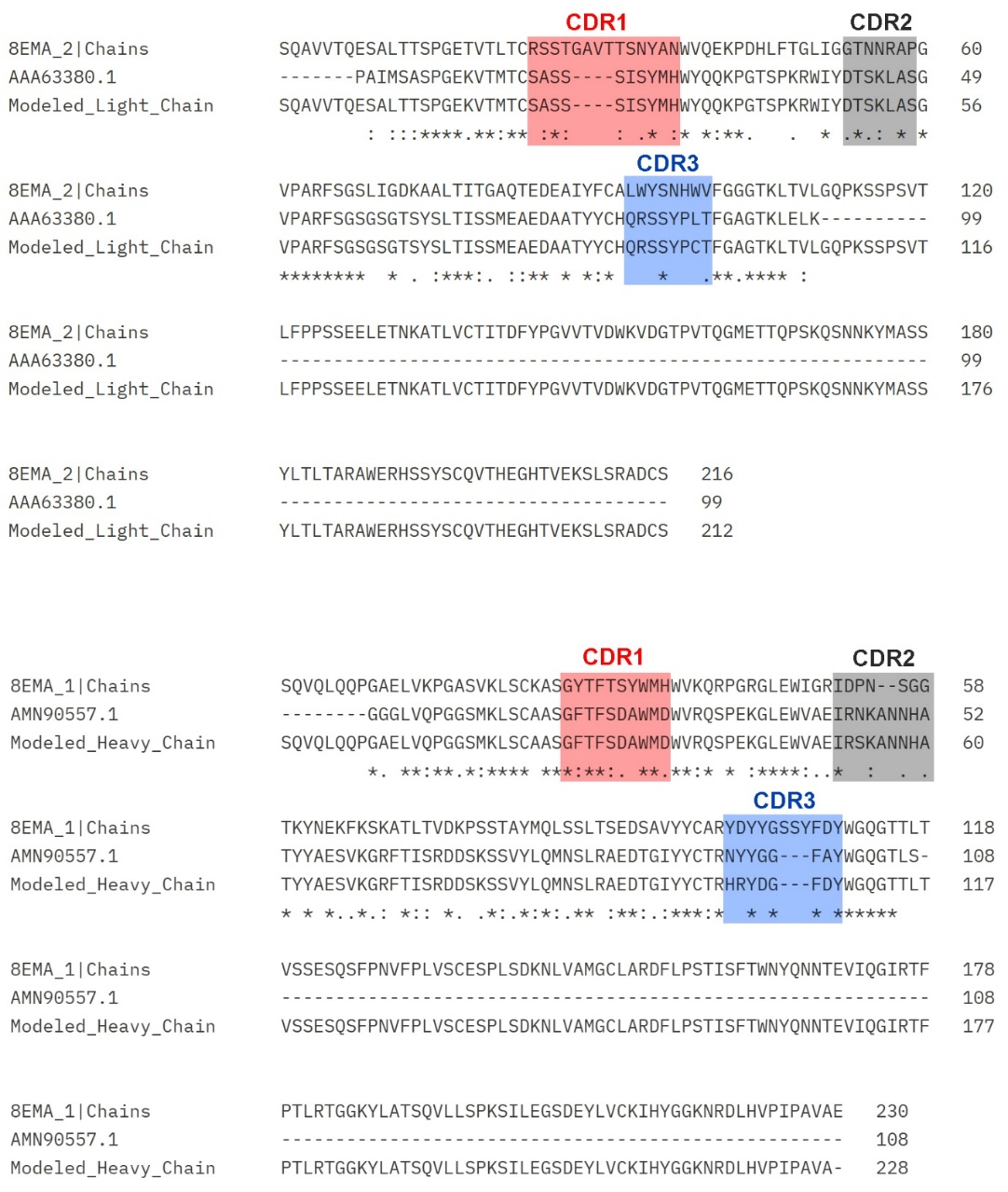


**SupplementaryFigure S1.** **Sequence alignment analysis to generate structural model of MAbCC5.** Multiple sequence alignments of mouse B cell receptor (PDB ID: 6EMA), AAA, light chain (GenBank: AAA63380.1), and heavy chain GenBank: (AMN90557.1) using Clustral Omega. The residues contributed to CDR1, CDR2, and CDR3 are highlighted in red, grey, and blue, respectively.

**1. Amino acid sequences used for modeling light chain of MAbCC5**

>8EMA_2|Chains C[auth L], D[auth R]|Anti-human Langerin 2G3 lambda chain|*Mus musculus* (10090)

SQAVVTQESALTTSPGETVTLTCRSSTGAVTTSNYANWVQEKPDHLFTGLIGGTNNRAPGVPARFSGSLIGDKAALTITGAQTEDEAIYFCALWYSNHWVFGGGTKLTVLGQPKSSPSVTLFPPSSEELETNKATLVCTITDFYPGVVTVDWKVDGTPVTQGMETTQPSKQSNNKYMASSYLTLTARAWERHSSYSCQVTHEGHTVEKSLSRADCS

>AAA63380.1 Ig kappa chain, partial [*Mus musculus*]

PAIMSASPGEKVTMTCSASSSISYMHWYQQKPGTSPKRWIYDTSKLASGVPARFSGSGSGTSYSLTISSMEAEDAATYYCHQRSSYPLTFGAGTKLELK

> Modeled_Light_Chain

SQAVVTQESALTTSPGEKVTMTCSASSSISYMHWYQQKPGTSPKRWIYDTSKLASGVPARFSGSGSGTSYSLTISSMEAEDAATYYCHQRSSYPCTFGAGTKLTVLGQPKSSPSVTLFPPSSEELETNKATLVCTITDFYPGVVTVDWKVDGTPVTQGMETTQPSKQSNNKYMASSYLTLTARAWERHSSYSCQVTHEGHTVEKSLSRADCS

**2. Amino acid sequences used for modeling heavy chain of MAbCC5**

>8EMA_1|Chains A, B|Isoform 2 of Immunoglobulin heavy constant mu|*Mus musculus* (10090)

SQVQLQQPGAELVKPGASVKLSCKASGYTFTSYWMHWVKQRPGRGLEWIGRIDPNSGGTKYNEKFKSKATLTVDKPSSTAYMQLSSLTSEDSAVYYCARYDYYGSSYFDYWGQGTTLTVSSESQSFPNVFPLVSCESPLSDKNLVAMGCLARDFLPSTISFTWNYQNNTEVIQGIRTFPTLRTGGKYLATSQVLLSPKSILEGSDEYLVCKIHYGGKNRDLHVPIPAVAE

>AMN90557.1 immunoglobulin heavy chain variable region, partial [*Mus musculus*]

GGGLVQPGGSMKLSCAASGFTFSDAWMDWVRQSPEKGLEWVAEIRNKANNHATYYAESVKGRFTISRDDS KSSVYLQMNSLRAEDTGIYYCTRNYYGGFAYWGQGTLS

> Modeled_Heavy_Chain

SQVQLQQPGAELVQPGGSMKLSCAASGFTFSDAWMDWVRQSPEKGLEWVAEIRSKANNHATYYAESVKGRFTISRDDSKSSVYLQMNSLRAEDTGIYYCTRHRYDGFDYWGQGTTLTVSSESQSFPNVFPLVSCESPLSDKNLVAMGCLARDFLPSTISFTWNYQNNTEVIQGIRTFPTLRTGGKYLATSQVLLSPKSILEGSDEYLVCKIHYGGKNRDLHVPIPAVA
